# The Working Memory Model and the relationship between immediate serial recall and immediate free recall

**DOI:** 10.1177/17470218241282093

**Published:** 2024-11-01

**Authors:** Geoff Ward, Philip C Beaman

**Affiliations:** 1 Department of Psychology, University of Essex, Colchester, UK; 2 School of Psychology and Clinical Language Sciences, University of Reading, Reading, UK

**Keywords:** Free recall, serial recall, primacy effect, recency effect, working memory, transposition errors

## Abstract

The effects of speech-based variables on the immediate serial recall (ISR) task constitute fundamental evidence underpinning the concept of the Phonological Loop component of Working Memory. Somewhat surprisingly, the Phonological Loop has yet to be applied to the immediate free recall (IFR) task although both tasks share similar memoranda and presentation methods. We believe that the separation of theories of ISR and IFR has contributed to the historical divergence between the Working Memory and Episodic Memory literature. We review more recent evidence showing that the two tasks are approached by participants in similar ways, with similar encoding and rehearsal strategies, and are similarly affected by manipulations of word length, phonological similarity, articulatory suppression/concurrent articulation, and irrelevant speech/sound. We present new analyses showing that the outputs of the two tasks share similar runs of successive items that include the first and last items– which we term start- and end-sequences, respectively—that the remaining residual items exhibit strong recency effects, and that start- and end-sequences impose constraints on output order that help account for error transposition gradients in ISR. Such analyses suggest that similar mechanisms might convey serial order information in the two tasks. We believe that recency effects are often under-appreciated in theories of ISR, and IFR mechanisms could generate error transpositions. We hope that our review and new analyses encourage greater theoretical integration between ISR and IFR and between the Working Memory and Episodic Memory literature.

## Introduction

It is difficult to overstate the importance of the *Working Memory Model* (WMM, Baddeley, 1986; Baddeley & Hitch, 1974, 2019) to the examination of immediate memory and its role in higher-order cognitive processes, such as reading, comprehension, reasoning, and learning (for an impressive list of applications, see Baddeley et al., 2021). The WMM is highly intuitive and readily understandable, has impressive longevity with relatively infrequent developments (most notably, Baddeley, 1986, 2000), and has served first as a pioneering framework, then a relatively stable leading account, and finally a point of departure for alternative theories (e.g., Andrade, 2001; Conway et al., 2008; Gathercole, 1996, 2001; Miyake & Shah, 1999).

This article focuses on the most developed component of the WMM, the Phonological Loop, which embodies the intuitive idea that we rapidly forget even small amounts of verbal information unless we actively maintain these items through rehearsal. The Phonological Loop was proposed to explain the effects of speech-based variables on the immediate serial recall (ISR) task and also to help explain the short-term retention of verbal material during higher order cognitive tasks such as reasoning, comprehension, and learning (Baddeley & Hitch, 1974), chess (Robbins et al., 1996), and task switching (Baddeley et al., 2001). Over time, a cognitive toolbox has been developed to explore the role of working memory in an impressive range of tasks and participant populations. While examining the effects of speech-based variables on the ISR task, this toolbox includes examining the effect of impeding the Phonological Loop by using a concurrent digit load and examining the relative impairments caused by concurrent articulation versus visuo-spatial tapping.

In this article, we address whether the Phonological Loop account of ISR could and / or should be extended to the highly related immediate free recall (IFR) task (for an earlier consideration of this issue, see Ward, 2001). We review recent evidence that encourages the theoretical integration of the two tasks, and we consider four issues that we believe must be addressed before the Phonological Loop model can be successfully applied to the IFR task including the role of rehearsal and the effects of speech-based variables in the two tasks, the contribution of episodic (long-term) memory to immediate recall in the two tasks, the importance of modality and recency effects in the two tasks, and the way in which serial position is represented. In our considerations, we argue that the magnitude of recency effects in ISR is often under-appreciated since, owing to earlier omissions in recall, recency items are often output too early to score as correct in conventional serial order scoring. By contrast, we show that in both tasks, participants often recall sequences of consecutively presented items that either initiate with the first list item (runs that we term *start-sequences*) and/or that culminate with the final list item (runs that we term *end-sequences*). We further show that given the known information inherent in start- and end sequences, any other recalled item will tend to be positioned at or close to its correct output position such that the benchmark locality constraint in ISR (the tendency for incorrectly ordered items to be recalled in neighbouring output positions) could arise in the absence of any further position information for these incorrectly ordered items. In this way, we suggest that the serial position effects and output orders in ISR and IFR may be generated using similar memory mechanisms, that the ISR data need not necessitate positional coding, and the similarities may encourage further theoretical integration of the two tasks. We argue that a speech-based verbal rehearsal mechanism, such as the Phonological Loop, could contribute to an integrated account of ISR and IFR and argue that this may be so, but only as an auxiliary mechanism supporting maintenance and retrieval from episodic long-term memory.

## The historical separation of IFR and ISR

One might think it surprising that the Phonological Loop has not yet been extended from the ISR task to the IFR task. Both tasks share highly similar methodologies: In each task, participants are presented with sequences of (typically verbal) stimuli, one at a time, and at the end of the list, participants must try to recall as many of the list items as they can in either the same order as they had been presented (ISR) or in any order that they wish (IFR). Both tasks also share a common theoretical heritage, providing classic empirical evidence that has been key to the development of the concept of a limited-capacity short-term memory store (STS), namely, the memory span limitations in ISR and the recency effect in IFR (the recall advantage for the last few list items). Despite these similarities, the Phonological Loop account of ISR (like many other accounts of short-term or working memory) has not as yet been applied to the related IFR task, whereas most classic and contemporary accounts of IFR (like many accounts of episodic memory) have not as yet been applied to the related ISR task.

As reviewed by Ward et al. (2010), there are at least three reasons for the historical divergence between the ISR (working memory) and the IFR (episodic memory) literature. First, when participants are asked to learn a 16-word list for free recall, the magnitude of the recency effect in free recall is unaffected by the concurrent requirement to maintain a 6-digit sequence for ISR (Baddeley & Hitch, 1974, 1977; Bhatarah et al., 2006). This finding appears to show that the recency effect in IFR and the memory span in ISR cannot both be attributed to the same STS because one would have expected catastrophic trade-offs between recency and ISR if the sequences of 6 digits and the last few words were underpinned by the same limited-capacity store. Thus, “it is suggested that working memory, which in other respects can be regarded as a modified STS, does not provide the basis for recency” (Baddeley & Hitch, 1974, p. 81) and “working memory is supposed to have both buffer-storage and control-processing functions, with recency explained by a separate mechanism” (Baddeley & Hitch, 1974, p. 82).

Second, early reviews suggested that ISR was affected by speech-based variables giving rise to the phonological similarity effect, the word length effect, the effects of articulatory suppression, and unattended or irrelevant speech^1^, whereas the recency effect in IFR was not particularly sensitive to these variables (e.g., Baddeley, 1976, p. 182). This again suggested that the mechanisms for maintaining and retrieving the items in ISR are different from those used to output the most recent items in IFR.

Finally, there are clear differences in the shapes of the serial position curves observed in classic ISR and IFR data sets. The serial position curves in IFR (e.g., Glanzer & Cunitz, 1966; Murdock, 1962; Postman & Phillips, 1965) are characterised by smaller primacy effects (the recall advantage for the items presented at the beginning of the list) and larger recency effects; whereas the serial position curves in ISR are characterised by larger primacy effects and reduced recency effects (e.g., Conrad & Hull, 1964; Drewnowski & Murdock, 1980; Jahnke, 1963). Theories of IFR have tended to focus on the mechanisms underpinning the recency effect (e.g., Beaman & Morton, 2000; Davelaar et al., 2005; Howard & Kahana, 2002; Lehman & Malmberg, 2013; Raaijmakers & Shiffrin, 1981; Tan & Ward, 2000), whereas theories of ISR have tended to focus on the mechanisms underpinning the primacy effect (e.g., Hurlstone, 2024; Lewandowsky & Farrell, 2008; Page & Norris, 1998).

The WMM is far from alone in explaining just one of the two immediate recall tasks. Many classic and contemporary theories of ISR do not also account for IFR (e.g., Baddeley, 1986, 2000, 2007, 2012; Baddeley & Hitch, 1974, 2019; Brown et al., 2000; Burgess & Hitch, 1992, 1999, 2006; Farrell & Lewandowsky, 2002, 2008; Henson, 1998, 2008; Logan & Cox, 2021; Nairne, 1990; Neath & Nairne, 1995; Oberauer & Lewandowsky, 2008; Page & Norris, 1998; Saint-Aubin et al., 2021). Similarly, many classic and contemporary accounts of IFR do not also account for ISR (e.g., Davelaar et al., 2005; Gillund & Shiffrin, 1984; Healey & Kahana, 2016; Howard & Kahana, 2002; Laming, 2006, 2008, 2009, 2010; Lehman & Malmberg, 2013; Lohnas et al., 2015; Polyn et al., 2009; Raaijmakers & Shiffrin, 1981; Sederberg et al., 2008; Tan & Ward, 2000).

However, a growing body of evidence suggests that results obtained from IFR and ISR may converge when the two tasks are examined using similar methods, list lengths, and scoring systems (Ward et al., 2010). Historically, classic studies of IFR have examined recall of longer lists of 10-40 words and have scored recalled words as correct irrespective of their order of recall (FR scoring). By contrast, classic studies of ISR have examined recall using shorter lists of 5-8 words and have scored recalled words as correct only if they are output in the same serial position as that in which they had been presented (SR scoring). It is possible that differences previously observed between the two tasks could reflect differences in list length and scoring systems—decisions taken by the experimenter—rather than a more fundamental difference between the memory mechanisms used to undertake the tasks by the participant. When the two tasks are examined under more similar conditions, more recent evidence suggests that there is a need for theoretical integration between the two tasks.

## The case for theoretical integration between ISR and IFR

Four existing lines of evidence support the case for integration. First, the two tasks are encoded and rehearsed in similar ways (Bhatarah et al., 2008, 2009; Grenfell-Essam & Ward, 2012). Bhatarah et al. (2008) presented three groups of participants with lists of eight words for immediate recall. One group of participants (pre-cued ISR) was told in advance that they would always be asked to recall the words in the same order as they had been presented. A second group of participants (pre-cued IFR) were told in advance that they would always be asked to recall the words in any order that they wished. A final group of participants were presented with lists of eight words for immediate recall but were only told after the last list item had been encoded (but prior to recall) that they would be asked to recall in either the same order (post-cued ISR) or recall in any order (post-cued IFR). Bhatarah et al. found that the shapes of the serial position curves were relatively unaffected by knowing the task in advance. Characteristic U-shaped serial position curves were observed in the two IFR conditions (plotted using FR scoring) that were very similar whether the test was expected and predictable (pre-cued IFR) or unpredictable (post-cued IFR). Similarly, extended primacy effects with little or no recency were observed in the two ISR conditions (using SR scoring) regardless of whether the task was known in advance (pre-cued ISR) or not (post-cued ISR). These findings were replicated by Bhatarah et al. (2009) who also showed that the patterns of rehearsals were broadly similar across the four conditions.

Second, there is a growing appreciation that both tasks show a tendency for forward-ordered recall, although this is not a formal task requirement in free recall (Bhatarah et al., 2008; Golomb et al., 2008; Howard & Kahana, 1999; Kahana, 1996; Klein et al., 2005; see also Beaman & Jones, 1998). Indeed, temporal contiguity occurs across a wide range of tasks, stimuli, and timescales in episodic memory (Healey et al., 2019).

**Figure 1.**
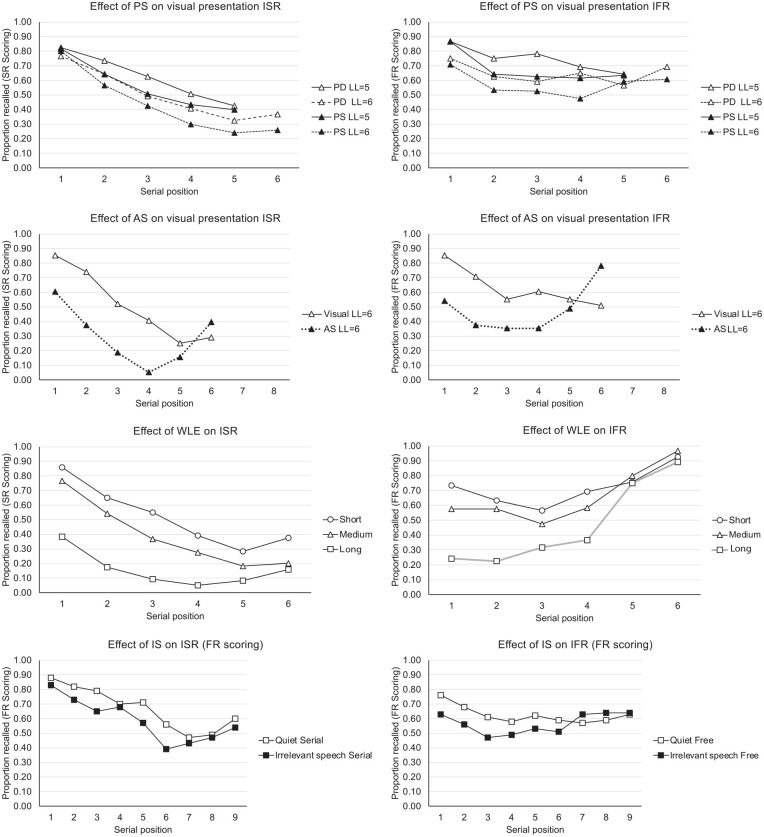
From top to bottom, comparison of the effects of Phonological Similarity (PS), Articulatory Suppression (AS), Word Length (WL) and irrelevant speech (IS) on Immediate serial recall (ISR, left hand panels) and Immediate Free Recall (IFR, right-hand panels). The phonological similarity data and articulatory suppression data are generated from data from Spurgeon et al. (2014, Experiments 2a, 2b) and Spurgeon et al. (2014, Experiment 1), respectively. The word length data are adapted with permission from Figure 7 of Bhatarah et al. (2009). The irrelevant speech/sound data are adapted with permission from Beaman and Jones (1998). Note: PS refers to Phonological Similarity (Phonologically-similar, PS and Phonologically-dissimilar, PD), AS refers to Articulatory Suppression (visual AS and visual silent), WL refers to Word Length (WL, short, medium and long words) and IS refers to Irrelevant Speech/Sound (Quiet, IS) on Immediate serial recall (ISR, left hand panels) and Immediate Free Recall (IFR, right-hand panels). LL refers to a specific List Length (selected from a wide range).

Third, there are similarities in the effects of a range of different variables on IFR and ISR, including presentation rate (Bhatarah et al., 2009), presentation modality (Grenfell-Essam et al., 2017), temporal isolation (Grenfell-Essam et al., 2019), and temporal grouping (Spurgeon et al., 2015). Figure 1 shows the effects on ISR and IFR of the four effects that are most commonly associated with the Phonological Loop: phonological similarity (Spurgeon et al., 2014), word length (Bhatarah et al., 2009), articulatory suppression (Spurgeon et al., 2014), and irrelevant speech (Beaman & Jones, 1998). In all cases, speech-based effects that are assumed to be the signatures of the Phonological loop in ISR are also observed in IFR. In both tasks, speech-based variables thought to affect the ability to rehearse (namely, articulatory suppression and word length) appear to have the greatest effect on the early serial positions.

Finally, these similarities increase when using the same list lengths and scoring systems. With short lists, participants tend to initiate recall with the first list item in both tasks and when they do, recall tends to proceed in forward order, resulting in elevated recall of early list items and reduced recency effects. For example, when presented with a short list of random words for IFR, such as “cat, house, fog, stairs,” there is a strong tendency for participants to recall the list in exactly the same order as presented, i.e., recall “cat, house, fog, stairs” even though forward-ordered recall is not a task requirement in IFR (Corballis, 1967; Grenfell-Essam & Ward, 2012; Neath & Crowder, 1996; Ward et al., 2010). With far longer lists, participants in both tasks find it hard to initiate recall with the first list item and instead initiate recall with one of the last four words, recall then tends to continue in forward order, resulting in extended recency effects and reduced primacy effects (Grenfell-Essam & Ward, 2012; Ward et al., 2010).

## Issues when applying the Phonological Loop account of ISR to IFR

If one accepts that IFR and ISR may be more similar than was once assumed, then how can theoretical integration be accomplished? Only a few theorists have tried to model both IFR and ISR within the same framework (Anderson et al., 1998; Brown et al., 2007; Farrell, 2012; Grossberg & Pearson, 2008). None of these computational models are attempts to implement the Phonological Loop construct and, of these, the model by Anderson et al. (1998) relies upon different processes (involving different parameters and different parameter values) for recalling items in IFR and ISR, and both the model by Anderson et al. (1998) and that of Grossberg and Pearson (2008) assume very different rehearsal patterns in the two tasks, an assumption that seems at odds with the data by Bhatarah et al. (2008, 2009) and Grenfell-Essam and Ward (2012), both of which suggest that the two tasks are encoded and rehearsed in similar ways. The models of Brown et al. (2007) and Farrell (2012) are more promising, in that they specifically attempt to unify short-term and episodic memory, although the former says little about output orders in recall, and neither account includes mechanisms for rehearsal, a mechanism central to the WMM. We will return to a more extended discussion of the Farrell (2012) model in a later section.

If we put aside integrative models from outside the broader working memory framework and take instead the Phonological Loop account of ISR as our starting point, then what issues must be addressed before it can contribute to an integrated account of ISR and IFR? In what follows we consider, in turn, the following four issues:

1. What is the nature of rehearsal and speech-based variables in immediate recall tasks?
2. What is the contribution of episodic (long-term) memory in these immediate recall tasks?
3. What is the nature of modality effects and recency effects in immediate recall?
4. How is serial order represented in the two tasks?

### What is the nature of rehearsal and speech-based variables in the recall of the two tasks?

Our first issue concerns the putative role of verbal rehearsal and the effects of speech-based variables on ISR and IFR (but for contrasting reviews on the causal role of rehearsal on immediate recall, see Lewandowsky & Oberauer, 2015; Oberauer, 2019; Ward, 2024). Phonological Loop theorists have championed the importance of speech-based variables in determining the memory span and accuracy in ISR. To many, it is highly intuitive that ISR should be linked with covert speech and verbal rehearsal and so be affected by factors such as phonological similarity, word length, irrelevant speech, and articulatory suppression. It is arguably one of the more impressive and coherent aspects of the WMM that it explains the interactions between the modality of presentation, articulatory suppression, and the phonological similarity and word length effects in ISR (Baddeley, 1986; Baddeley et al., 1984; although see also Hughes, 2024; D. M. Jones et al., 2004). The importance of speech-based variables in determining accuracy in IFR as well as ISR (see Figure 1) suggests a common theoretical interpretation; to the extent that the Phonological Loop can account for the ISR data, it seems reasonable that it should also be applied to the IFR data. Despite this, no theory of IFR has to our knowledge tried to model the effects of these speech-based variables, although the effects of phonological similarity (Spurgeon et al., 2014), irrelevant speech (Beaman & Jones, 1998), word length (Bhatarah et al., 2009), and articulatory suppression (Bhatarah et al., 2009; Spurgeon et al., 2014) are all found in both tasks, and similar rehearsal patterns are seen in IFR and ISR (Bhatarah et al., 2009).

Numerous studies have also shown that differences in memory span between individuals and between different types of stimuli reflect differences in rehearsal rates between participants (e.g., Hulme et al., 1984; Naveh-Benjamin & Ayres, 1986) and between the speech rates of the stimuli (e.g., Baddeley et al., 1975; Ellis & Hennelly, 1980; Murray & Jones, 2002; Schweickert & Boruff, 1986; Standing et al., 1980), respectively. In IFR, there is considerable evidence that the probability of recall is also a positive function of the number of rehearsals (Rundus, 1971), the recency of the rehearsals (Brodie & Murdock, 1977), and the distribution of the rehearsals (Modigliani & Hedges, 1987), with all three variables most likely to be important (see Tan & Ward, 2000; Ward & Tan, 2023). Rehearsal can also reorder the presented stimuli in IFR as rehearsal and reminding leading to subjective re-organisation (Ward & Tan, 2023). Verbal or articulatory rehearsal therefore likely represents a common element in the two tasks.

Although performance in IFR and ISR tends to benefit from greater opportunities to rehearse, it is important not to overstate the role of rehearsal in these tasks. It is well-established that not all differences in memory span between different stimulus materials can be attributed to differences in rehearsal rates, but rather spans are additionally affected by long-term lexical knowledge concerning words (e.g., Hulme et al., 1991) and the co-occurrence of words (G. Jones & Macken, 2018), including things such as word frequency effects (e.g., Hulme et al., 1997), concreteness effects (Walker & Hulme, 1999), orthographic and phonological neighbourhood effects (Roodenrys et al., 2002), and semantic factors (e.g., Poirier & Saint-Aubin, 1995; Saint-Aubin & Poirier, 1999). In addition, recent evidence suggests that the retention of order in ISR through rehearsal is most effective if rehearsal is limited to subspan sequences of stimuli (Barrouillet et al., 2021; Jarrold, 2017). If one encourages and instructs participants to rehearse greater sequence lengths than would have been spontaneously generated then ISR accuracy does not improve (Souza & Oberauer, 2018, 2020). Critically, both ISR and IFR can be performed in situations where verbal rehearsal is less likely (albeit performance is sometimes reduced), such as with faster presentation rates (e.g., Oberauer, 2022; Tan & Ward, 2008), with articulatory suppression (e.g., Grenfell-Essam et al., 2013; Oberauer, 2022; Spurgeon et al., 2014), or with non-verbal stimuli (Cortis et al., 2015; D. M. Jones et al., 1995; Ward et al., 2005).

Moreover, it is important to note that rehearsal is often assumed to serve a different function in ISR and IFR. In the WMM, a primary function of rehearsal is to refresh the activation of phonological codes of the presented items in the Phonological Store that would otherwise suffer trace decay if left unrehearsed. That is, the function of rehearsal is to offset a negative effect associated with time (trace decay). In contrast, theories of IFR assume a more positive function for rehearsal. Yes, theories of IFR may assume that an unrehearsed item may become less accessible following a delay (due to changes in temporal distinctiveness or context discrimination, or increased competitiveness of other list items), but rehearsing an item in theories of IFR is generally thought to strengthen the associations between itself and the current list in LTM (Raaijmakers & Shiffrin, 1981), the associations between itself and other co-rehearsed items (Raaijmakers & Shiffrin, 1981), or to increase its later accessibility from episodic memory by providing multiple different retrieval routes and multiple different contexts (including more recent contexts) in which it was encoded (Tan & Ward, 2000). Arguably, rehearsal must do more than simply maintain the original level of activation. Increased rehearsals and repetitions of stimuli increase the probability of recall of those stimuli in both tasks as evidenced by higher accuracies with slower presentation rates in IFR (Glanzer & Cunitz, 1966; Murdock, 1962; Roberts, 1972; Tan & Ward, 2000) and in ISR (Oberauer, 2022; Tan & Ward, 2008).

### What is the contribution of episodic (long-term) memory in these immediate recall tasks?

In a revision of the WMM, Baddeley (2000) proposed the need to incorporate an Episodic Buffer. The Episodic Buffer was envisaged as a limited-capacity temporary storage system that holds episodes of integrated information across space and potentially over time from a variety of sources and codes. The revision was proposed following a discussion of many of the limitations of rehearsal also raised by us in the preceding paragraphs, and importantly, to confront the need to relate WM with LTM. In the revision, the contributions of the episodic buffer and episodic long-term memory to ISR were not specified, other than to suggest it acted as a “back-up store” in those situations (e.g., conditions with visual presentation and articulatory suppression) where the operation of the Phonological Loop was unlikely. Phenomena such as the Hebb repetition effect (Hebb, 1961), the superior recall of sequences of items that have previously been presented as part of a to-be-recalled list, and the effects of long-term (e.g., lexical) knowledge provide evidence for one kind of contribution of long-term memory to ISR but these are generally considered as reflecting the build-up of knowledge over time rather than a direct episodic recollection (Baddeley et al., 1998). However, we believe that some contribution of episodic memory must surely also be expected in ISR beyond this given that participants can readily recall list items from prior lists in a delayed free recall test (e.g., Loaiza & McCabe, 2012; McCabe, 2008) and participants benefit from their repetition in spin lists (e.g., Kahana et al., 2010; Solway et al., 2012) and serial learning and multi-trial free recall learning tasks (e.g., Klein et al., 2005; Waugh, 1961).

By contrast, all theories of IFR specify the contribution of episodic long-term memory during encoding and retrieval (Brown et al., 2007; Crowder, 1993; Greene, 1992; Howard & Kahana, 2002; Lohnas et al., 2015; Polyn et al., 2009). Indeed, many theories of IFR assume that *all* encoding and retrieval is from episodic memory, but many additionally propose the need for recall from a STS (e.g., Atkinson & Shiffrin, 1971; Davelaar et al., 2005; Lehman & Malmberg, 2013; Raaijmakers & Shiffrin, 1981; Unsworth & Engle, 2007). For those who assume that IFR is a two-component task (see e.g., Baddeley, 1986; Glanzer, 1972), the recall of words presented at early and middle serial positions are assumed to be retrieved from long-term episodic memory (and so are selectively affected by variables such as presentation rate, word frequency, and list length), whereas the most recent items are assumed to be directly retrieved from a separate STS (and so are selectively affected by variables such as the modality of presentation and the presence of a filled delay). What is lacking therefore is a more-specified account of what, if any, role is played by the Episodic Buffer and episodic LTM in WMM accounts of ISR (and IFR).

### What is the nature of modality and recency effects in immediate recall?

The Phonological Loop is predominantly a theory of rehearsal and forward-ordered recall and as such it does not readily account for the modality effect in ISR, nor recency effects, more generally. The modality effect refers to the enhanced recall of items presented auditorily rather than visually within the recency portion of the serial position curve (e.g., Beaman, 2002; Beaman & Morton, 2000; Conrad & Hull, 1964; Crowder & Morton, 1969; Grenfell-Essam et al., 2017). Although one would think that an explanation of the modality effect should be explicable within the WMM framework, Baddeley, (1986, p. 87) stated that “while a complete model of the working memory system would most certainly incorporate this interesting and productive area of research, the model has at present little to say on these phenomena.” To our minds, some of the intuitive appeal of the WMM is lost by its inability to capture the recall advantage of the last few items presented. Modality effects occur with both serial and free recall, although the magnitude of the auditory advantage is typically observed to be greater in serial recall (near-perfect recall for the final item) but a smaller auditory advantage tends to be extended across far more serial positions in free recall. These apparent discrepancies can be explained by the difference in list lengths that are typically used. As shown by Grenfell-Essam et al. (2017), the magnitudes and the extents of the modality effects in the two tasks tend to converge when the list lengths are equated, and an “inverted modality effect,” the superior performance for visual presentation at earlier points in the list (Beaman, 2002), is also observed in both tasks (Grenfell-Essam et al., 2017). Thus, while a complete explanation of the modality effect must encompass a variety of data not included here (e.g., the effects of a post-stimulus suffix and the nature of lip-read and non-verbal recency; Campbell & Dodd, 1980; Greene & Samuel, 1986), there is no *a priori* reason to dismiss a common account covering both serial and free recall.

An explanation of the recency effect is also critical for any extension of the WMM to IFR. We have already mentioned prior research that showed that the magnitude of the recency effect was relatively unaffected by a concurrent digit span task (Baddeley & Hitch, 1974, 1977), studies that had suggested that recency lies outside the WMM. Subsequent studies further suggested that recency effects occur across a wide range of timescales (Baddeley, 1986, chapter 7; Baddeley, 2007, chapter 6; da Costa Pinto & Baddeley, 1991; Hitch & Ferguson, 1991), consistent with the ratio rule of Glenberg and Swanson (1986). As we will see, the ideas of long-term recency through temporal distinctiveness (Glenberg and Swanson, 1986) and discrimination of fluctuating temporal context (Estes, 1955; Mensink & Raaijmakers, 1989) have been highly influential in contemporary accounts of IFR (Brown et al., 2007; Howard & Kahana, 2002). Although Baddeley and Hitch (1993) later suggested an implicit priming interpretation of these effects, the idea that recency is delivered by an explicit retrieval strategy operating on presented stimuli provides a promising starting point for the extension of the WMM to IFR. Once a list of words had been presented, participants could flexibly elect to use either of two separate retrieval cues: an explicit retrieval cue that would generate recency from episodic long-term memory or a separate cue to initiate forward-ordered recall from the start of the list using the Phonological Loop. If this were the case, then one might consider why participants would not also use the recency-based cue to assist in ISR? As we will see, the magnitude of the recency effect is underappreciated in most theories and data sets concerning ISR.

### How is serial order represented in the two tasks?

Finally, perhaps the biggest challenge for extending the Phonological Loop to IFR arises when one considers how serial position information is represented across the two tasks. The WMM (Baddeley, 1986; Baddeley & Hitch, 1974) provided qualitative accounts of working memory phenomena without specifying the mechanisms for retaining serial order information. Subsequent computational models of ISR have been developed to model these working memory phenomena, with many directly inspired by the Phonological Loop. Henson (1998, 2001) proposed three categories of proposed serial order mechanisms: ordinal theories, positional theories, and associative chaining theories. Many theories of ISR incorporate multiple mechanisms to deliver all the working memory phenomena (for more detailed review of theories of ISR, see Lewandowsky & Farrell, 2008; Hurlstone et al., 2014; Hurlstone, 2024; Osth & Hurlstone, 2023), but these serial order mechanisms are primarily proposed to produce forward-ordered primacy effects. However, we would like to argue that an integrated account of ISR and IFR must be capable of generating both primacy and recency effects, and there must be scope, even with ISR, to allow participants to demonstrate their undoubted cognitive flexibility and output in different orders using task-appropriate retrieval strategies. For example, the bulk of the data on ISR has been obtained by asking participants to recall a list in the order in which it was presented, starting with the first item. However, studies have also looked at backward serial recall using an ISR paradigm (e.g., Li & Lewandowsky, 1995), and it is straightforward to show that participants are capable of initiating ordered recall (and, presumably, rehearsal) from an arbitrary given point if required to do so (Beaman, 2002).

Computational models of the Phonological Loop have tended to use ordinal and/ or positional mechanisms for serial order. Ordinal theories of serial recall assume that earlier list items are encoded more strongly than later list items (Farrell & Lewandowsky, 2002; Grossberg & Pearson, 2008; Page & Norris, 1998) resulting in a primacy gradient extending across the early serial positions. At each point in serial recall, the most activated item is selected, output, and then that response is suppressed, before the next most activated item is selected, and so on. This process gives rise to extended primacy effects and one-item recency (due to the edge effect). Noise is added at response selection, and the resulting errors are most typically transpositions where a later stimulus item becomes more highly activated than its immediately preceding list item, and so is output too soon, followed by *fill in*, the recall of the next highly activated item which tends to be its transposed partner. Errors tend to be transpositions between near-neighbouring list items (the locality constraint), but omissions and item errors are also observed, their frequencies increase across output positions (due to decay or interference).

Positional theories assume that each stimulus item is associated with an abstract representation of its list position or temporal context. At test, it is assumed that participants can retrieve the positional marker or reset the temporal context to that associated with the first list item, and the positional marker is assumed to evolve during a test, iteratively cueing successive list positions. In some models, the context evolves with new events (Burgess & Hitch, 1992; Farrell, 2006; Lewandowsky & Farrell, 2008) whereas in others, the context is more closely associated with time (Brown et al., 2000, 2007; Burgess & Hitch, 1999, 2006; Hartley et al., 2016). The list position is normally referenced by its distance from the start of the list, but it can also be referenced by its distance from the end of the list (Henson, 1998)—which must be an unrealistic assumption if the list length varies markedly and unpredictably across successive lists (see Grenfell-Essam & Ward, 2012; below). Many positional theories also assume primacy gradients (Brown et al., 2000; Burgess & Hitch, 1999; Henson, 1998; Lewandowsky & Farrell, 2008).

Computational models of the Phonological Loop have tended to reject associative chaining models of serial order. Simple associative chaining models (e.g., Lewandowsky & Murdock, 1989) assume that each presented item is associated with its predecessor. Compound chaining models (Murdock, 1993, 1995; Solway et al., 2012) assume forward and backward associations between both adjacent and non-adjacent items; the strengths of the associations decrease across different positions. At test, some additional mechanism is required to access the first list item such as a start of list cue (Lewandowsky & Murdock, 1989), but the representation of the list could be encoded across all items (Logan, 2021; Logan & Cox, 2021, 2023) and used at test. There are perceived difficulties in how simple associative chaining models can generate the locality constraint, how they deal with lists containing repeated stimuli, and how they can model participants’ ability to recover from error. Moreover, it is often assumed that associative chaining models should have particular difficulty in recalling lists that alternate between phonological similar and dissimilar list items (Baddeley, 1968; Henson et al., 1996), but these difficulties can be overcome if (like many other accounts of ISR) one assumes separate layers dealing with order and items (see Osth & Hurlstone, 2023).

By contrast, while all theories of IFR explain the bowed serial position curves, not all theories of IFR satisfactorily explain output order. In dual-store theories of free recall, it is assumed that participants output first the contents of STS (the order in which the items are output is rarely stated) before long-term memory is searched using the list context as a cue, after which additional retrieved items can also be used as cues. Words that are rehearsed during the study will increase their associative strength with the list context, and co-rehearsed items will increase their inter-item associative strength. Primacy effects in dual-store accounts of IFR are typically explained by increased rehearsal of the early list items (Raaijmaakers & Shiffrin, 1981; Rundus, 1971). However, recall is probabilistic and there is no guarantee that the recency items or the primacy items will be recalled from STS or LTS in forward serial order. Other accounts predict primacy effects and recency effects based on the increased temporal distinctiveness of the first and particularly the last items (Brown et al., 2007), but there is again no clear mechanism proposed to order output in IFR, and a positional code is necessary to additionally account for ISR. Finally, some accounts of IFR assume that the start of list context (Davelaar et al., 2005; Metcalfe & Murdock, 1981) or “Get Ready” warning signal (Laming, 1999, 2010) are encoded and retrieved at test to give access to the start of the list. Latency data show that initiating recall with the first list item is far slower than initiating recall with one of the more recent list items (Laming, 1999; Osth et al., 2021; Osth & Farrell, 2019), which can be taken as evidence that initiating recall with the first item or later items involve different retrieval decisions. Thus, with the exception of the position coding of perhaps the first list item, many of these theories of IFR have not used ordinal or positional coding to code serial positions.

The accounts of IFR that provide the most detailed accounts of output orders are the Context Maintenance and Retrieval (CMR) theories (e.g., Healey & Kahana, 2016; Kahana, 2020; Lohnas et al., 2015; Polyn et al., 2009) derived from the Temporal Context Model (TCM, Howard & Kahana, 2002). In these models, successive stimuli are associated with a temporal context that evolves throughout the presentation of the list. Unlike earlier models that had assumed that temporal context randomly drifts with time (e.g., Estes, 1955; Glenberg & Swanson, 1986; Mensink & Raaijmakers, 1988), in TCM and its CMR variants, it is the pre-experimental associations to presented stimuli that are retrieved and used to drive the changes in the temporal context. In this way, later stimulus items are encoded with temporal contexts that accommodate a recency-weighted function of recently experienced list items.

These models readily explain recency effects in IFR: The end of the list context is used to cue recall and owing to its greater overlap with the contexts associated with more recent items, the end of list context is most likely to cue one of the most recent list items. These models also assume temporal regularities in the output order: It is assumed that the retrieved context of a recalled item is used as a cue and so neighbouring items are most likely recalled, with a heightened tendency to recall the next list item (asymmetric temporal contiguity effect). Some primacy can be incorporated by assuming that the early list items are more strongly attended to and/or are more strongly encoded. However, most experimental data sets modelled by TCM and its variants are of relatively long lists during which participants must perform an orienting task, such that the primacy effect is markedly reduced relative to the recency effect. Some CMR-inspired models have allowed for additional context representation to also code the encoding task (Polyn et al., 2009), the start of the list (Kragel et al., 2015; Morton & Polyn, 2016), or the list context (Healey & Wahlheim, 2024). Interestingly, a list context is also used in recent CMR-inspired models of serial recall (Logan, 2021; Logan & Cox, 2021, 2023), and these CMR-inspired models offer an alternative starting point for the theoretical integration of the two tasks. We shall return to these alternative accounts in a section towards the end of the article.

We have already reviewed prior work suggesting that words are rehearsed and encoded in similar ways in IFR and ISR, such that the different serial position curves typical of the two tasks must largely reflect differences in retrieval strategies, output interference, and scoring systems. In particular, we wish to argue that participants must have far greater accessibility to the most recent list items in ISR immediately at the test, notwithstanding that strong and extended recency effects are not always observed in ISR serial position curves. We argue that recency effects are greatly reduced following the output interference of earlier items (Beaman, 2002; Bhatarah et al., 2008, 2009; Cowan et al., 2002; Grenfell-Essam & Ward, 2012; Lewandowsky et al., 2009; Oberauer, 2003; Tan & Ward, 2007; Ward & Tan, 2019) and that recency effects are reduced using SR scoring, because SR scoring systems penalise as incorrect the recall of terminal runs of recency items in recall sequences containing one or more omissions. Thus, we wish to argue that whatever serial order mechanism(s) are proposed for IFR and ISR, they must be capable of generating both primacy and recency effects and it should be possible for participants to output in different orders using task-appropriate retrieval strategies.

**Figure 2.**
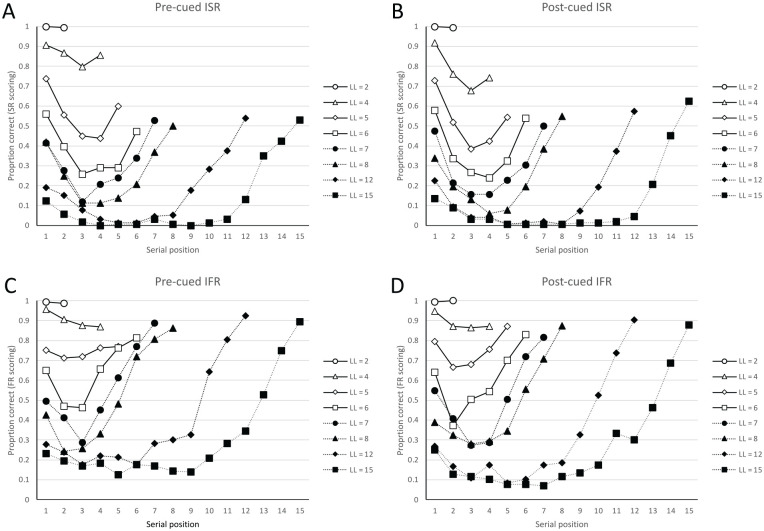
Data from Grenfell-Essam and Ward (2012, Experiment 3). Figure adapted from Grenfell-Essam and Ward (2012). *Note*: Figure adapted with permission from Figures 12 and 13 of Grenfell-Essam and Ward (2012). Serial position curves for lists of between 2 and 12 words are presented for immediate serial recall (ISR, top panels) and immediate free recall (IFR, lower panels). The left-hand panels show data for participants who always knew the method of testing before encoding (i.e., the task is pre-cued); the right-hand panels show data for participants who only knew the method of testing after encoding, immediately prior to retrieval (i.e., the task is post-cued).

To illustrate these points, consider the ISR and IFR data of Grenfell-Essam and Ward (2012, Experiment 3) presented in Figure 2 in which three groups of participants were presented with lists of between 2 and 15 words for immediate recall. The words were presented individually on a computer screen at a rate of 1 word per second and were read aloud by the participants. One group (pre-cued ISR) always knew that they would be required to perform ISR, and their lists were always prefixed and suffixed with the cue “same.” A second group (pre-cued IFR) always knew that they would be required to perform IFR, and their lists were always prefixed and suffixed with the cue “any.” A third group encoded the list of items without knowing the required test. Their lists were always prefixed by the uninformative cue “??????”, and the task requirements on that trial were revealed immediately prior to recall by the suffix cue “same” indicating to recall in the same order (post-cued ISR) or by the suffix cue “any” indicating to recall in any order (post-cued IFR). After the post-cue, the screen changed to reveal a grid containing the same number of numbered rows as there were words on the current trial and helped inform participants of the list length of that trial. Participants always wrote their recalls in response sheets which contained numbered grids of 15 lines. With ISR instructions, participants could only recall in forward order, and participants in all conditions vocalised their written responses as they recalled. A word was scored as correct if it was output at any grid position in IFR (FR scoring) and was scored as correct only if it was output in the same grid position as its serial position in ISR (SR scoring).

The left-hand panels of Figure 2 show the serial position curves of the pre-cued ISR condition (using SR scoring, Figure 2A) and pre-cued IFR conditions (using FR scoring, Figure 2C). It is immediately apparent that accuracy in both tasks reduces with longer lists, and that with task-specific scoring, there is more extended primacy with ISR and more extended recency with IFR. In part, the reduced recency in ISR relative to IFR reflects output interference: in ISR these terminal items can only be recalled after the recall of any earlier list items, whereas in IFR they can be output first. However, when one looks at the panels showing the serial position curves of a pre-cued ISR task, one might also be struck by the finding that as list length increases the recency effects in ISR become more extended than are typically observed using conventional SR scoring. Thus, given the opportunity then it is possible to show recency extending over several serial positions even with ISR. Conversely, the serial position cues of an IFR task show more evidence of primacy at shorter list lengths in terms of a more obvious uplift for the first 1 to 2 items than is typically seen in free recall using longer lists.

The right-hand panels of Figure 2 show the serial position curves for the post-cued ISR (using SR scoring, Figure 2B) and post-cued IFR conditions (using FR scoring, Figure 2D). The words in these two post-cued conditions must have been encoded in the same way because participants could not reliably anticipate the instructed task prior to recall. Critically, those differences between the ISR and IFR tasks observable in the pre-cued tasks remain in the post-cued conditions when scored traditionally. Thus, the serial position curves of the post-cued ISR condition resembled those from the corresponding pre-cued ISR condition (using SR scoring), and the serial position curves of the post-cued IFR condition resembled those from the corresponding pre-cued IFR condition (using FR scoring).

Participants’ prior knowledge of the output requirements—which might prompt different encoding and maintenance strategies—cannot therefore be the important factor in creating the differences between IFR and ISR; rather these differences must reflect the action of the experimenter in instructing different recall orders and applying different scoring criteria. This replicates and extends earlier data by Dalezman (1976) showing that post-list instructions on the order in which to prioritise the recall items in an otherwise “free” recall task changes the shape of the serial position curve, primarily by boosting primacy and reducing recency when subjects were asked to recall the beginning of the list first. Similarly, the recall of earlier recalled items tends to be enhanced in versions of ISR, in which participants are instructed to initiate recall at particular points of the list (e.g., Beaman, 2002; Cowan et al., 2002).

## Start- and end-sequences in IFR and ISR

Until this point, this article has largely reviewed prior existing work that encourages the theoretical integration of IFR and ISR. We have argued that to the extent that the Phonological Loop model provides an adequate account of ISR, it should also be able to be applied to IFR data. One might reasonably ask how extending the model from ISR to IFR might inform what types of serial order information are strictly necessary to account for the primacy and recency effects that are observed in Figure 2.

In this section, we present new analyses re-examining the serial order information contained within the Grenfell-Essam and Ward (2012) data. Our starting point is that the WMM and many theories of ISR are not well-placed to generate the large and extended recency effects observed in our ISR (and IFR) data, while theories of IFR are not obviously well placed to output sequences of 5 to 7 items in correct serial order. How do participants performing ISR know that the 14th presented item in a 15-item list should be positioned in grid position 14?

One integrated solution to generate recency effects in ISR (as well as IFR) would be to assume that each presented stimulus item is associated with a continuously-evolving temporal context (e.g., Davelaar et al., 2005; Glenberg, 1984, 1987; Glenberg & Swanson, 1986; Mensink & Raaijmakers, 1988, 1989; Tan & Ward, 2000); and assume further that the retrieved pre-experimental associations of that item help drive the evolution of the temporal context (Healey & Kahana, 2016; Howard & Kahana, 2002; Kahana, 2020; Lohnas et al., 2015; Polyn et al., 2009). An evolving temporal context encoded at learning, retrieved at recall, and used to cue item representations are common to many models of both serial and free recall but in serial recall, it is constrained so that it reinstantiates the start of the list context at retrieval. If the end-of-list context is used as a retrieval cue, then it is most likely to cue a recent item (e.g., n-2, n-1, or n), which if retrieved, could itself be used to cue successive list items. Through the principles of first recency and then temporal contiguity, participants could find that they have retrieved one, two, or three consecutively-presented end-of-list items terminating with the last list item, i.e., an **end-sequence**, which they could then allocate to the last one, two, or three list positions, respectively.

An integrated solution for generating primacy effects in IFR (as well as ISR) would be to assume that participants at the test are able to cue the start of the list (cf. Brown et al., 2000; Burgess & Hitch, 1999; Farrell, 2012; Logan, 2021; Logan & Cox, 2021, 2023). If so, then the retrieved context could be used to retrieve successive items, creating a run of one, two, three, or more consecutively presented stimulus items starting with the first list item, i.e., a **start-sequence**. A focus on start- and end-sequences is consistent with early conclusions from recall studies that stronger or unique retrieval cues are associated with the beginning and ends of lists (Dalezman, 1976; Tulving, 1968). At slower rates, forward-ordered recall might be augmented further by the active maintenance of cumulative forward-ordered rehearsal in the Phonological Loop.

**Figure 3.**
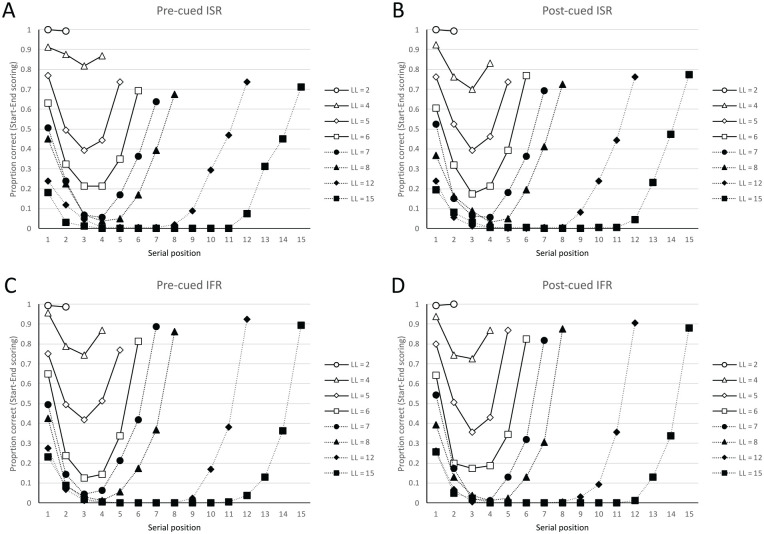
Data from Grenfell-Essam and Ward (2012, Experiment 3) using start-end scoring. A word is only scored as correct if it is output as part of a start-sequence (a run of consecutive recalls starting with the first presented word) or as part of an endsequence (a run of consecutive recalls ending with the last presented word). The upper panels show data from immediate serial (ISR) when the task is known (Figure 3A) or not known (Figure 3B) prior to encoding. Similarly, the lower panels show data from immediate free recall (IFR) when the task is known (Figure 3C) or not known (Figure 3D) prior to encoding.

To clarify our novel start- and end-sequence scoring procedure, let us represent an 8-item list of presented words with the 8 letters, ABCDEFGH. Suppose that in a test of IFR, participants recalled the following four sequences of recalls: FGHCABG, HGABCE, ABCEGH, GFAC. One way to measure the serial order information present at recall is to score these recalled sequences in terms of what we call start-sequences and end-sequences. In our new analyses, a start-sequence is defined as a run of recalls from consecutive serial positions in the original list, initiating with the first presented word, A. Similarly, an end-sequence is defined as a run of recalls of words from consecutive serial positions in the original list, terminating with the last presented word, H. If one scores start-sequences in **bold** and end-sequences in *italics*, then the first sequence of recalls could be expressed as *FGH*C**AB**G (an end-sequence of three, and a start-sequence of two), with the remaining three sequences of recall attempts rendered as *H*G**ABC**E, **ABC**FE*GH*, GF**A**C. Figure 3 applies the scoring of start- and end-sequences to the Grenfell-Essam and Ward (2012) data. To be clear, in our new reanalyses, the recalled sequences refer to the temporally ordered sequence of vocalised words rather than the assigned grid positions at which the recalled words were written.

As Figure 3 shows, there are even more striking similarities between the four serial position curves when the new start- and end-sequence scoring system is applied to both tasks. Importantly, the forward serial order information (start-sequences) that one might assume would be conveyed by the Phonological Loop in the ISR data appears to be similarly present in the IFR data. In addition, there is considerable recency present in both tasks as evidenced by the similar end-sequences in both IFR and ISR.

**Figure 4.**
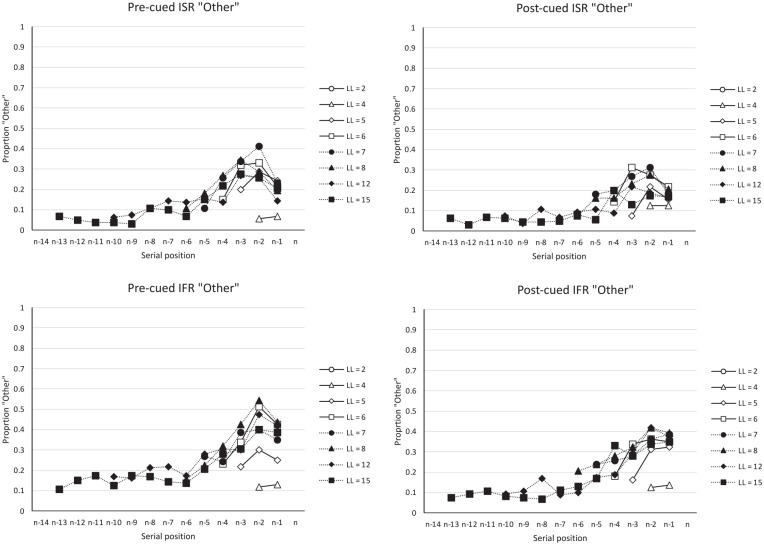
Data from Grenfell-Essam and Ward (2012, Experiment 3) plotting the proportion of “Other” words (words that were recalled that were not in a start-sequence or an end-sequence). These data have been recency-justified such that more recent serial positions are aligned to the right of the panels.

The start- and end-sequence analyses performed upon the Grenfell-Essam and Ward (2012) data suggest that theories of IFR and ISR need to be able to generate such start- and end-sequences as recall-entities in their own right, albeit ones of varying size and scope. One might then reasonably ask, what are the characteristics of the “Other” items (those recalled items not output in start- or end-sequences), and how might they be recalled? Figure 4 shows the residual serial position curves which plot the proportion of words recalled as “Other” items in the four conditions. Unlike most serial position curves which are primacy-justified, plotting serial position 1 on the far left-hand side of the serial position curve, the serial position curves in Figure 4 have been recency-justified, such that the last list item in each list length are presented on the right-hand side of each panel. The figure shows that for both IFR and ISR there is little residual primacy and considerable residual recency. That is, the primacy effect observed in Figure 2 appears to come almost entirely from the start-sequences; when these are removed from the serial position curves as in Figure 4, the “Other” items show little recall advantage for items near the beginning of the list. By contrast, Figure 4 shows that the recency effect arises not only from the end-sequences but when the end-sequences are removed, there remains a more general recall advantage for items less distant from the end of the list, a finding more consistent with recency-based accounts of IFR and episodic (long-term) memory. This finding could, however, potentially be explained if final list items are not only more accessible than middle items but have less positional certainty than the primacy items (cf. Henson, 1998).

## Using start- and end-sequences with more standard ISR data sets

At this point, one might wonder whether our findings are limited to the Grenfell-Essam and Ward (2012) data set, which could be considered somewhat unusual in varying the list length and recalling written serial recall in lined grids. In this section, we re-examine more standard ISR data from the impressively large data set from Osth and Dennis (2015) who presented four groups of almost 100 participants with 62 experimental trials consisting of lists of 6 words presented at a rate of 1.25s per word. A series of three question marks (???) acted as a recall cue for participants to recall the list items in forward serial order by typing in each word followed by the enter key which cleared each response. Participants typed “done” to conclude their recall. The four between-subjects conditions of Osth and Dennis (2015) were the Open condition (6128 trials) in which the stimuli were sampled from an open set (the six words were always different on each trial), the Blanks condition (6186 trials), which also used an open set of stimuli but the participants were encouraged to type “blank” to signal an omission, the Closed condition (6198 trials) in which the stimuli were sampled from a closed set (the same six words were always presented in different random orders on each trial; the set of six words were randomly sampled for each participant from the Open set stimulus pool), and the Reconstruction condition (5797 trials), in which an open set of stimuli and a reconstruction of order test were used (at test, the six list items were re-presented in a new random order and remained in view while participants performed recall).

In our second reanalyses, we re-analyse data from these more standard ISR methodologies to examine the extent to which the serial position curves are also determined by the start- and end-sequences. We are interested in the length of the start sequences and the length of the end sequences. How much recency is apparent in these more standard ISR data sets and why is there so little recency in the standard serial position curves with ISR scoring? Specifically, we reanalysed the recall sequences on each trial of each participant for the four conditions of Osth and Dennis (2015), and in each recalled sequence, we categorised the recalls as being part of a start-sequence, as being part of an end-sequence, or not in either type of sequence which we categorise as “other.” Table 1 shows the different combinations of start- and end-sequences in the data in the four conditions. First, there were different proportions of trials in which the recalled sequences were completely correct, “123456,” the proportions increasing with the ease of recalling the items. Thus, in the Reconstruction of Order and Closed conditions, where the list items are known at test or were constant from trial to trial, these proportions of completely correct sequences were 0.472 and 0.383, respectively; whereas when the items were unknown and varied from trial to trial, these proportions of completely correct sequences were 0.209 and 0.152, respectively.

**Table 1.** Immediate serial recall (ISR) data of Osth and Dennis (2015).

		Length of end-sequence					
Group	Length of start sequence	No end	End “6”	End “56”	End “456”	End “3456”	End “23456”
Closed ISR	no start	232	83	39	20	31	25
	start “1”	392	135	74	52	31	0
	start “12”	412	171	97	58	2	
	start “123”	448	211	93	0		
	Start “1234”	588	244	5			
	start “12345”	331	7				
	start “123456”	2347					
Open ISR	no start	377	86	50	31	30	30
	start “1”	569	171	60	32	60	0
	start “12”	654	209	95	69	0	
	start “123”	595	190	163	1		
	Start “1234”	733	405	5			
	start “12345”	626	0	2			
	start “123456”	943					
Blanks	no start	367	84	34	17	19	38
	start “1”	542	112	40	26	51	1
	start “12”	612	159	65	75	0	1
	start “123”	529	179	139	1	1	
	start “1234”	793	427	1			
	start “12345”	589	2				
	start “123456”	1294					
Open RoO	no start	197	63	26	23	19	31
	start “1”	286	134	79	74	50	0
	start “12”	349	196	111	34	0	
	start “123”	321	270	49	1	3	
	start “1234”	485	117	4			
	start “12345”	127	8	1			
	start “123456”	2739					

The frequency distribution of sequences of recalls containing different combinations of start- and end-sequences.

Note: ISR refers to Immediate Serial Recall; RoO refers to Reconstruction of Order. In the Open, Blanks and RoO groups, six new words were sampled without replacement on each trial. In the closed group, six words from the stimulus set were randomly sampled without replacement on the first trial, and then repeatedly reshuffled on all subsequent trials. In the Blanks condition, participants were encouraged to type “blank” to indicate an omission.

**Figure 5.**
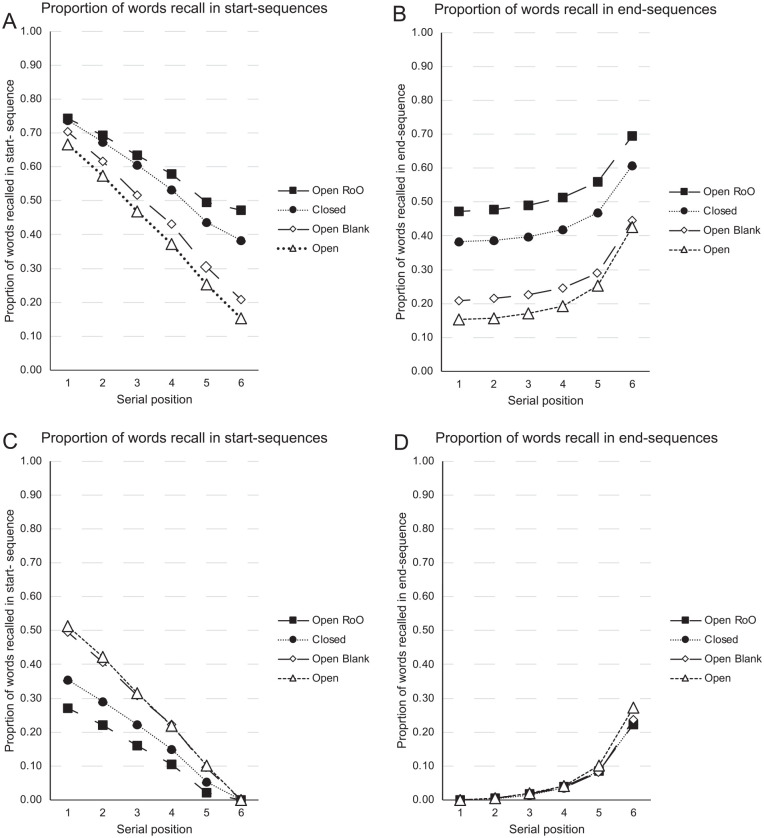
Data from Osth and Dennis (2015). The proportion of words that were correctly recalled as part of a start-sequence (Panel 5A) or end-sequence (Panel 5B) when the completely correctly recalled sequences were included. The proportion of words that were correctly recalled as part of a start-sequence (Panel 5 C) or end-sequence (Panel 5D) when the completely correctly recalled sequences were excluded. Note: ISR refers to Immediate Serial Recall; RoO refers to Reconstruction of Order. In the Open, Open Blanks, and RoO groups, six new words were sampled without replacement on each trial. In the closed, six words from the stimulus set were randomly sampled without replacement on the first trial, and then repeatedly reshuffled on all subsequent trials. In the Open Blanks condition, participants were encouraged to type “blank” to indicate an omission.

Table 1 also shows that a good proportion of trials in each group contained different combinations of incomplete “start-” and “end-sequences.” Figure 5A and B shows the proportion of words recalled as part of start-sequences and end-sequences, respectively, when the proportions include the trials in which the recalled sequences were completely correct; Figure 5 C and D shows the proportion of words recalled as part of start-sequences and end-sequences, respectively, when the proportions do not include the trials in which the recalled sequences were completely correct. As can be seen, there is considerable primacy and recency in these ISR data, with the primacy effect from the start-sequences being more sensitive to the level of support for the recall of the items across the four groups (Figure 5C) than the recency in the end-sequences (Figure 5D).

**Figure 6.**
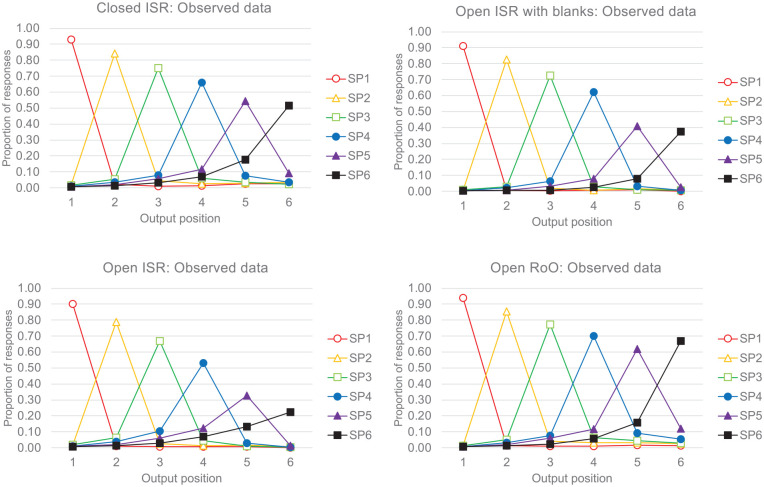
Data from Osth and Dennis (2015). The proportions of words presented at each serial position (SP) recalled at output positions 1 to 6. The peaks in each distribution show that words were most often recalled in the correct order.

Given the ISR instructions, we assumed that participants would first output the start sequence, then recall the “Other” items in a random order, before ending their recall with the end sequence. Of interest was the patterns of order errors that were generated using this procedure. In studies of ISR, it is typical to plot error transposition gradients that show the probability of recalling, in each of the different possible output positions, a word that had been presented in a given input serial position. Figure 6 shows the error transposition gradients for the four conditions of Osth and Dennis (2015). Each panel shows the proportion of recalled items as a function of their input serial position (different coloured lines) across the different output positions (x-axes). The peaks in these distributions show that the presented words were most often correctly recalled in their correct output position: e.g., the third presented item was most often recalled third, the fourth presented item was most often recalled fourth, and so on. Were one to join up the peaks of these distributions, then this provides the serial position curves. While there is clear evidence of extended primacy effects, there is little or no evidence of recency in ISR using correct in-position scoring (i.e., little or no recall advantage of outputting the sixth item in the sixth output position compared with outputting the fifth item in the fifth output position), despite the evidence for end-sequences in these data (Figure 5 and Table 1).

As is typical, words that were recalled in incorrect output positions were typically recalled at near-neighbouring locations (the locality constraint), a finding that has been argued to support ordinal or positional models. For example, Henson et al. (1996) concluded:

“The present study has shown how detailed analysis of patterns of errors can shed considerable light on the nature of the mechanisms required in a successful model of immediate serial recall. *The locality constraint* [the preponderance of errors which are transpositions of nearby items] *shows that errors arise through mechanisms beyond random guessing.*” (Henson et al, p.110, italic emphasis added)

**Figure 7.**
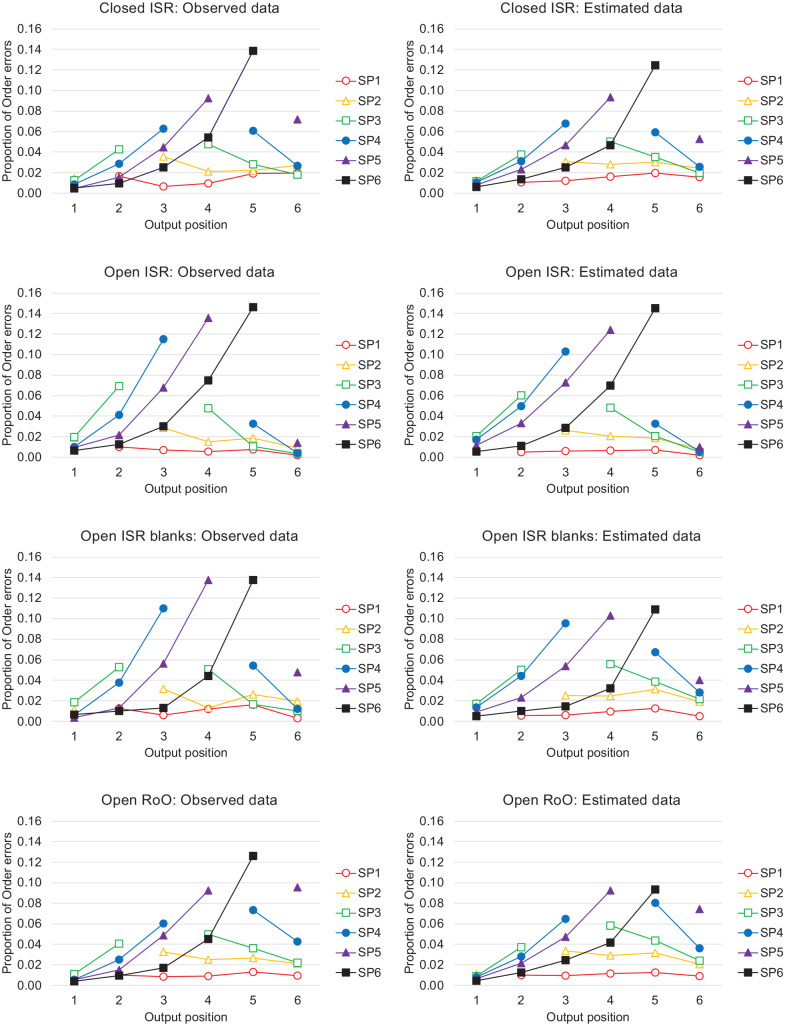
Data from Osth and Dennis (2015). The proportion of order errors in the observed data (left-hand panels) and the estimated data (right-hand panels). The only serial position information assumed in the estimated data is that inferred from start- and end-sequences.

The patterns of incorrectly ordered recalls are difficult to examine in the panels of Figure 6 because the proportions of the incorrect responses are small relative to the proportions of the correct responses. These distributions of incorrectly ordered responses are more easily observed in the left-hand panels of Figure 7, which do not show the correct recalls, but plot the distributions of incorrectly ordered recalls as proportions of the total numbers of order errors in that condition. As shown in the left-hand panels of Figure 7, for each presented input serial position, the proportion of incorrectly ordered recalls is greatest at the nearest neighbouring output positions, clearly illustrating the locality constraint.

Of interest is the representation of serial order that is necessary to generate these error gradients. Why is the word presented in the fourth serial position more often recalled in output positions 3 or 5, rather than at more distant output positions, 2 or 6? Does this suggest that ordinal or positional coding of all items is necessary for ISR data? If so, this might present a barrier to the theoretical integration of ISR and IFR because ordinal or positional coding is rarely assumed in theories of IFR.

As an expository device, we consider an extreme alternative possibility, that participants know *nothing* about the serial position of items that are not recalled as part of a start- or end sequence. Our analyses will therefore show how much order information is strictly necessary in the recall of 6-item lists for serial recall and which types of theories of ISR and IFR could potentially explain these data.

For each recalled sequence in the Osth and Dennis (2015) data, we categorised the recalled words as (i) being part of a start-sequence, (ii) being part of an end-sequence, or (iii) being an “Other” item. Note that “Other” items therefore include any recalled word that was not part of a start- or end-sequence that was recalled in the correct or incorrect output position, any list items that were repeated at output (repetitions), and any non-list items that had been presented on previous trials (prior-list intrusions) or had not been presented on any previous trial (extra-list intrusions). We assumed that any start-sequence items would be output first, any end-sequence items would be output last, and any “Other” list items would be randomly allocated to intervening output positions. The right-hand panels of Figure 7 show the proportion of output errors generated by our “start + guess + end” estimates. Perhaps surprisingly, our estimated distributions resemble quite closely the patterns of observed errors in the ISR data, although we assumed that there was no additional serial position information contained within the output sequences beyond that contained in the start- and end-sequences.

There are two main reasons why our estimated distributions of errors closely resemble the observed error distributions. First, participants often outputted fewer responses than there were words, and in such cases, any end-sequences that were recalled would necessarily be output prematurely in earlier output positions. Second, although the “Other” items were assumed not to possess any inherent serial position information, their output positions were nevertheless constrained to lie *between* start and end sequences. If one accepts the argument that start-sequences and end-sequences constrain recall for the mid-list items in a similar way, then something very like the random guessing that was dismissed by Henson et al. (1996) *a priori* becomes worthy of more serious consideration^2^.

The importance of this demonstration is that we have shown that reasonably plausible error transposition gradients can be generated in ISR even in the absence of positional information associated with these order errors, just so long as the output order of recalled “Other” items is constrained by known start-sequences and end-sequences. As mentioned earlier, very few theories of free recall assume that items are associated with detailed serial position information, and so the removal of this constraint widens the range of possible theories of serial order that could explain serial position phenomena in ISR and IFR. Most primacy effects in IFR arise through start-sequences, if one removes the start- and end-sequences from IFR data, then the resultant serial position curves show extended recency, but little residual primacy (e.g., Figure 4). A model of IFR that generates start- and end-sequences might not only correctly generate the serial position curve in IFR, but make considerable progress in generating the serial position curves and error transpositions in ISR.

Before continuing, it is important to acknowledge a number of nuances and limitations that arise from our analyses. First, we acknowledge that the observed start- and end-sequences that are present in participants’ recall data are unlikely to exactly reflect the start- and end-sequences known by the participant at the time of the test. Indeed, if our hypotheses are correct, then we must assume that the observed start- and end-sequences which we are starting from are most likely inflated since they are likely to include the lucky positioning of “Other” items that through guessing were correctly assigned to extend start- or end-sequences. It is also possible that through some other cause of failure (e.g., typing error of B to B’) an item in a known sequence, ABCD, may be mistyped, AB’CD, such that the observed start sequence appears truncated, deflating the estimated sequence length. We further acknowledge that a generative model would help clarify the sufficiency of this approach.

Second, although our analyses could be taken as an important counterpoint to ordinal and positional accounts of the locality constraint, we do not rule out the possibility that some or all of the start- and end-sequences and transpositions gradients were generated by ordinal or positional codes, nor that some of the start- and end-sequences and transpositions gradients arise through guessing. Indeed, assuming that either (i) all transposition errors are caused by a confusion of positional cues, or that (ii) no transposition errors are caused by a confusion of positional cues could be considered extreme views. Nonetheless, the former is implicitly endorsed by any model of serial recall which does not include a (possibly metacognitive) guessing component, which is the majority of connectionist models, and our analyses provides an existence-proof that transpositions gradients could arise even in the absence of (more or less precise) positional information, given the constraints of start- and end-sequences.

## What are the benefits of separate start- and end sequences?

The use of different retrieval cues to try to initiate recall of separate start- and end-sequences is consistent with the finding that participants tend to initiate IFR with either the first list item or one of the last four list items (Ward et al., 2010). Implementing these two cues in different orders would allow participants the flexibility to perform IFR (typically end-cue then start-cue) or ISR (necessarily start-cue then end-cue) when instructions are post-cued, immediately prior to test (Bhatarah et al., 2008, 2009; Grenfell-Essam & Ward, 2012; Ward & Tan, 2019). Separate retrieval cues are also consistent with the latency data (Osth et al., 2021; Osth & Farrell, 2019) which shows that serial position 1 would be very unlikely to be ever output first based on any competitive race between items but must instead be chosen via a separate decision process.

Separate cues generating start- and end-sequences may help explain why the start and end of a list serve as anchors in serial learning and multi-trial free recall learning studies. The use of separate start-of-list and end-of-list retrieval cues offers a possible way to output some items when one cue fails entirely (such as when a start-of-list cue fails to access any items at increasing list lengths) or when the end-of-list cue fails (such as when a filled retention interval is inserted after the last list item). Since the end-of-list sequence is only generated at retrieval, there is no concern about how one encodes an end-of-list marker with widely varying and unpredictable list lengths (cf. Henson, 1998), and the generated end-sequences are relative to the end of the list and not based on input serial position from the start of the list (Henson, 1999).

A further benefit is that our analyses show that the Error Transposition gradients arise as an emergent property of the separate start- and end-sequences and need not be generated by an additional mechanism. Again, the suggestion that there are no other mechanisms for coding order is an extreme position, but one which might prove fruitful if appraised in conjunction with other considerations. For example, the original version of the feature model (Nairne, 1990) was successful in showing phonological confusion errors when list items shared (phonologically) similar features but did not show the correct pattern of errors without the addition of a stage in which order information was explicitly considered, and perturbation of such order cues was allowed, with cues more likely to drift or “perturb” to a nearby serial position (Neath, 1999). The reason why error patterns in Nairne’s (1990) original feature model were not correct was that each item was recalled independent of all the others, with the only constraint on recall being an increasing reluctance to recall any individual item more than once, so, in fact, error patterns within the original model were not random, they were systematically incorrect. However, if recall is constrained by knowledge of what has already been output, or by consideration of what is about to be output, then our analyses show that the choice between the remaining possibilities becomes more limited.

## Start- and end sequences and the WMM

This article considers whether the Phonological Loop could and / or should be extended from the ISR to the IFR task. Our review and new analyses suggest that there are far more similarities than differences between ISR and IFR, and we argue that the Phonological Loop should be extended to account for both ISR and IFR data. We have shown that speech-based variables, which are traditionally considered to be evidence for the involvement of the Phonological Loop in ISR show similar affects also in IFR. We have shown that in both tasks there are start-sequences and end-sequences, we suggest that there may be separate retrieval strategies to cue the start and the end of the list, and we have provided existence-proof that any non-sequenced “other” words that are recalled can be recalled at output positions that are close to the correct position (the locality constraint) even when no additional position information is assumed.

In terms of the WMM, we suggest that a verbal rehearsal mechanism may augment start sequences. Supplementary Material A1 confirms that the mean lengths of start-sequences are affected by many variables thought to affect rehearsal in the Phonological Loop. The mean length of a start-sequence decreases with articulatory suppression (Spurgeon et al., 2014), and decreases with word length (Bhatarah et al., 2009), and access to the start of the list decreases with increasing list length (see also Ward et al., 2010). However, it is critical to point out that if the Phonological Loop is considered to be involved in the generation and maintenance of start-sequences, it should be posited to also generate start-sequences in IFR.

By contrast, Supplementary Material A2 shows the mean length of end-sequences is far less affected by these variables, again, in both IFR and ISR. This suggests that the Phonological Loop concept may be less well-suited to explaining participants’ ability to cue the end of the list, and the generation of end-sequences. The end-sequences and extended recency effects are relatively unaffected by rehearsal, may be used more often with longer lists, and they occur even when the list length varies widely and unpredictably from list to list, ruling out end list position markers that are encoded with the stimuli (Henson, 1998). By acknowledging the roles of recency and episodic long-term memory more generally in ISR, it is potentially possible to preserve the importance of phonological loop variables on primacy effects and start-sequences while extending the WMM to IFR.

## Interpreting start- and end-sequences

The Phonological Loop and the WMM have until recently been largely agnostic with respect to the mechanism used to model serial position information and there are a wide range of possible approaches one could look to when exploring the mechanisms for serial order that could generate the start-sequences and end-sequences necessary for an integrated account of ISR and IFR.

One approach would be to start with existing theories of ISR. These include ordinal and positional accounts favoured by existing formal models inspired by the Phonological Loop model of serial recall (e.g., Burgess & Hitch, 1992, 1999, 2006; Henson, 1998; Henson et al., 1996; Page & Norris, 1998). These mechanisms could readily generate Error Transposition gradients by assuming that each item is associated with position or order information and that a common type of confusion that can arise at retrieval is in the incorrect positioning of items in neighbouring output positions Although valid, ordinal and positional theories of serial order tend to give rise to primacy effects, but our reanalyses of ISR and IFR data suggest that there also exist end-sequences and extended recency effects. Recency and end-sequences could be generated by associating stimuli with start- and end-position markers at encoding (Henson, 1998, 1999), but this only seems plausible with known list lengths.

A second approach would be to start with existing theories of IFR. Our novel reanalyses reconceptualise what serial order information is strictly necessary to generate IFR and ISR patterns of data. Rather than assume that all list items are encoded with respect to more or less precise position coding, our reanalyses suggest that many of the serial recall phenomena could be captured by IFR mechanisms, if only they could generate start sequences. The leading, most established accounts of IFR are derived from retrieved-context theories of episodic memory (Healey & Kahana, 2016; Howard & Kahana, 2002; Kahana, 2020; Lohnas et al., 2015; Polyn et al., 2009) that embody the principles of recency and temporal contiguity (Kahana et al., 2024). These models assume that items are associated with gradually evolving temporal context; the temporal context is assumed to evolve in part through the retrieval of pre-experimental semantic associations of the study items, such that the temporal contexts associated with later items contains a recency-weighted function of the contexts of earlier-presented items. Most simulations of these theories result in extended recency effects and strong temporal contiguity effects, but relatively weak primacy effects, and so it has been unclear, until recently, whether these types of models could generate sufficient primacy or generate the apparently intricate pattern of error transpositions observed in ISR. However, in the last five years, there has been growing interest and progress in using TCM-inspired models to model ISR (e.g., CRU, Logan, 2021; Logan & Cox, 2021, 2023). In the CRU model, the list context is represented within the temporal context that is associated with each item (enabling it to be used as a start cue) and the temporal context evolves over time, such that the end of list context has the potential to be used as an end-of list context. Unfortunately, CRU has yet to be applied to IFR. An alternative possibility is to incorporate a start of list context cue into the evolving temporal context allowing a CMR-variant (cf. PEPPR, Healey & Wahlheim, 2024; sCMR, Lohnas, 2023) to strategically cue the start or the end of the list with different retrieval cues. As yet, PEPPR has not been applied to ISR, but sCMR is a nascent attempt to integrate IFR and ISR. It should also be noted that no variant of TCM or CMR has as yet incorporated rehearsal mechanisms, so it is difficult to see how these accounts would deal with the effects of rehearsal and the phonological loop variables on ISR and IFR.

A third approach is to start with accounts that already integrate ISR and IFR. Of these, the account by Farrell (2012) offers the most detailed account of the changes in output orders that are observed in IFR and ISR of lists of different lengths. The Farrell (2012) model shows how a serially-ordered short-term or working memory mechanism—albeit not a deliberate attempt to implement the Phonological Loop construct—might account not only for serial recall but also for free recall data. Farrell (2012) assumes that a continuously presented list of items is spontaneously parsed by participants into one or more temporal groups. Individual items are associated with temporal context using Hebbian association, but unlike many other formal models of ISR and IFR, it assumes that the temporal context is hierarchically organised, with contexts organised into lists, lists organised into groups, and within-groups organised by within-group positions. The Farrell model combines many of the core mechanisms commonly found in formal models of serial recall with concepts from the free recall literature, such as output interference (Dalezman, 1976) and a stopping criterion at retrieval (Dougherty et al., 2014) to successfully model first ISR and then IFR data. To recall an item, participants must first explicitly retrieve that item’s group. Accessing the current group is straightforward, but accessing earlier groups is far more difficult and may lead to retrieval failure. Once a group has been successfully accessed, recall proceeds in a forward direction, commencing with the first item in the currently accessed group. Farrell (2012, pp. 241–242) shows that a model that includes a specific ordering element implemented via the context vectors not only reproduces patterns of order recall in a serial recall, as expected, but simulates memory for items in free recall uncorrelated with measures of retained order information (Input-Output correspondence or I-O scores). Critically, the Farrell model offers the flexibility to generate a forward-ordered start-sequences, by cueing for the first group context, which if successful will tend to generate a primacy-initial run of items, and offers the flexibility to generate end-sequences, by cueing with the current context, to generate a terminal run of list items commencing with the first item in the current group. The Farrell (2012) model is currently the best-published integrated account of IFR and ISR, but it contains multiple mechanisms for generating serial position information. Our work questions whether all these mechanisms are strictly necessary.

Yet another starting point would have been to focus on the feature characteristics of our stimuli in an attempt to explain the modality effect. The integrated account that we have sketched out currently says little or nothing about how a modality effect arises because there is nothing intrinsic to end-sequences *per se* to necessitate such a thing, albeit a TCM-inspired account of the modality effect has recently been proposed (Pazdera & Kahana, 2023). One candidate starting point is the feature model (Nairne, 1988, 1990, 2002; Neath, 2000; Neath & Nairne, 1995), which assumes that stimuli are represented by vectors of feature values. Auditory stimuli are assumed to be more richly encoded than silently-read visual stimuli and so are encoded with a greater number of features. The modality effect emerges because successive list items are assumed to overwrite the features of those immediately preceding them. Since the last list item benefits from not being overwritten, there is a one-item recency effect, which is greater in auditory lists. In fact, as suggested earlier, the magnitude and the extent of the modality effect varies with the list length and is present in both ISR (e.g., Conrad & Hull, 1964) and IFR (e.g., Murdock & Walker, 1969). In both tasks, the size of the recall advantage is smaller but spread over many terminal serial positions with longer lists and is larger but limited to just a single list item with shorter lists (Grenfell-Essam et al., 2017). The Grenfell-Essam et al. data show that the magnitude of the modality effect is far greater when participants initiate their recall from the start of the list and reduced when recall initiates at the end of the list, suggesting that the modality effect may arise because the recency items are far more resistant to output interference when they were read aloud or spoken to participants. These data are consistent with an earlier report by Beaman and Morton (2000) examining free recall only. In Beaman and Morton’s (2000) data, with the exception of auditory recency for the very final item, recency was largely dependent upon the appearance of end sequences within the free recall protocol. This could be because the effectiveness of an end-of-list cue is increased if features in the more richly encoded recency items have not at test been subject to interference from the silently generated recalled prior items.

More recently, the so-called Revised Feature Model (Cyr et al., 2022; Gionet et al., 2022; Saint-Aubin et al., 2023) accounts for the production effect in IFR and ISR by similarly assuming that read aloud items are encoded with more features than visual silent items. Unlike the original Feature Model, it assumes that the overwriting effects are spread over a number of prior items, allowing for extended recency effects. More importantly, it also assumes that early list items benefit from rehearsal—as we suggested for our start sequences—and further assumes that rehearsal is impeded by reading aloud the list items, giving rise to inverse modality effects (the recall advantage of visual items on earlier list items, Beaman, 2002; Grenfell-Essam et al., 2017; Macken et al., 2016).

A final starting point would be based on the perceptual-gestural framework advocated by D. M. Jones and colleagues (D. M. Jones et al., 2004, 2006; D. M. Jones & Macken, 2018). The key defining feature of this approach is the suggestion that verbal short-term memory phenomena should be reconceptualised as perceptual objects subject to control processes directed towards particular goals. This approach has much in common with other, neuroimaging-inspired, considerations that working memory might comprise a reactivation of the original perceptual representations of the objects maintained in a coherent form and distinct from ongoing perception by frontal lobe control processes. In our terms, a sequence (either a start-sequence or an end-sequence) could be such a perceptual object, with the goals defined by the experimenter-given instructions and the control processes counting start- and end-retrieval cues (and random guessing) within their number. Once again, an articulatory-rehearsal process is common to both—appearing within the D. M. Jones and Macken (2018) framework as a gestural component. Where we differ from D. M. Jones and Macken (2018) is in their rejection of the language of traditional concepts such as memory and forgetting—whether or not an end-sequence is best viewed as a perceptual object, it should be firmly embedded within—and relatable to—episodic (long-term) memory to speak to the voluminous literature on this task.

## Summary and conclusion

In this article, we have argued that the WMM should be extended from ISR to IFR. We have reviewed prior evidence that suggests that ISR and IFR tasks are more similar than once thought, including that the two tasks are similarly affected by speech-based, Phonological Loop variables. We discussed some of the dilemmas faced by the WMM in addressing an integration of ISR and IFR. Although the WMM appears well-placed to explain the effects of speech-based factors and rehearsal in the two tasks, there remains uncertainty as to how the WMM accounts for recency effects and modality effects, how the WMM interacts with episodic long-term memory, and how the WMM accounts for serial position effects. In our new analyses, we have shown that the output orders in both IFR and ISR contain important runs of consecutively presented items that initiate with the first list item (start-sequences) and culminate with the last list item (end-sequences). We believe that end-sequences and recency effects, more generally, are under-appreciated in many theories of ISR, whereas the generation of start-sequences and primacy effects, more generally, are under-appreciated in many theories of IFR. Moreover, we argue that a knowledge of start- and end-sequences may be sufficient to constrain the location of other words recalled, such that plausible error transposition gradients may be generated without recourse to further serial position information, a finding that may reduce the difficulties for theories of IFR to be extended to ISR data. Thus, we believe that the WMM would benefit from embracing these issues, broadening its scope in explaining a wider range of immediate memory tasks and phenomena, and specifying the relationship between WMM and episodic long-term memory.

Finally, one of the reviewers of this paper questioned how our analyses inform the functionality of memory. That is, using Baddeley’s (1988) phrase, there must be something a memory system is “for.” Although highly speculative, we believe that the functionality (and intuitive appeal) of the WMM could only be further increased by more fully integrating recency effects (including end-sequences): It is self-evidently important to situate events in context, and to have heightened accessibility to what has recently occurred in particular contexts (at a range of timescales). It is possible that start-sequences may assist with speech- and motor-planning, which when combined with phonological awareness and development could be used in speech and language comprehension and production, or vocabulary learning devices (Baddeley et al., 1998). Thus, the WMM in general and the Phonological Loop, in particular, would benefit from being extended to IFR (and other immediate memory tasks), would benefit from a greater acknowledgement of the role of recency (and modality) in the two tasks, and would benefit from a more precisely defined relationship between working memory and episodic long-term memory in immediate memory tasks.

## Supplemental Material

sj-docx-1-qjp-10.1177_17470218241282093 – Supplemental material for The Working Memory Model and the relationship between immediate serial recall and immediate free recall Supplemental material, sj-docx-1-qjp-10.1177_17470218241282093 for The Working Memory Model and the relationship between immediate serial recall and immediate free recall by Geoff Ward and Philip C Beaman in Quarterly Journal of Experimental Psychology

## Notes

1. In later writings, articulatory suppression and irrelevant or unattended speech effects are often referred to using the more theoretically neutral terms “concurrent articulation” and “irrelevant sound” effects (e.g., Beaman & Jones, 1997). Here, we deliberately use the original terms to emphasise the assumed links to speech processing.

2. It is worth noting some models already implement the benefit of a known end-sequence in serial recall to a limited extent. For example, the Primacy Model of Page and Norris (1998) simulates the modality effect within serial recall by assuming Precategorical Acoustic Storage (PAS; Crowder & Morton, 1969) of the final item. This guarantees near-perfect recall of that item and a “trickle-back” effect across the penultimate and antepenultimate items as, because the final item is known, it is no longer competing for recall at these positions. This exclusion of the final item for consideration prior to the end of the list results in superior recall performance not only for that item itself, but also across the final 2-3 serial positions for auditory lists (see also Beaman & Morton, 2000).

## References

[bibr1-17470218241282093] Anderson J. R. Bothell D. Lebiere C. Matessa M. (1998). An integrated theory of list memory. Journal of Memory and Language, 38(4), 341–380. 10.1006/jmla.1997.2553

[bibr2-17470218241282093] Andrade J. (Ed.). (2001). Working memory in perspective. Psychology Press.

[bibr3-17470218241282093] Atkinson R. C. Shiffrin R. M. (1971). The control of short-term memory. Scientific American, 225(2), 82–91. https://www.jstor.org/stable/24922803 5089457 10.1038/scientificamerican0871-82

[bibr4-17470218241282093] Baddeley A. D. (1968). How does acoustic similarity influence short-term memory? The Quarterly Journal of Experimental Psychology, 20(3), 249–264. 5683764 10.1080/14640746808400159

[bibr5-17470218241282093] Baddeley A. D. (1976). The psychology of memory. Basic Books Inc.

[bibr6-17470218241282093] Baddeley A. D. (1986). Working memory. Clarendon Press.

[bibr7-17470218241282093] Baddeley A. D. (1988). But what the hell is it for? In Gruneberg M. M. Morris P. E. Sykes R. N. (Eds.), Practical aspects of memory: Current research and issues: Vol. 1. Memory in everyday life (pp. 3–18). John Wiley & Sons.

[bibr8-17470218241282093] Baddeley A. D. (2000). The episodic buffer: A new component of working memory? Trends in Cognitive Sciences, 4, 417–423. 10.1016/S1364-6613(00)01538-2 11058819

[bibr9-17470218241282093] Baddeley A. D. (2007). Working memory, thought, and act (Vol. 45). Oxford University Press.

[bibr10-17470218241282093] Baddeley A. D. (2012). Working memory: Theories, models, and controversies. Annual Review of Psychology, 63, 1–29. 10.1146/annurev-psych-120710-100422 21961947

[bibr11-17470218241282093] Baddeley A. D. Chincotta D. Adlam A. (2001). Working memory and the control of action: Evidence from task switching. Journal of Experimental Psychology: General, 130(4), 641–657. 11757873

[bibr12-17470218241282093] Baddeley A. D. Gathercole S. Papagno C. (1998). The phonological loop as a language learning device. Psychological Review, 105(1), 158–173. 9450375 10.1037/0033-295x.105.1.158

[bibr13-17470218241282093] Baddeley A. D. Hitch G. J. (1974). Working memory. The Psychology of Learning and Motivation, 8, 47–89.

[bibr14-17470218241282093] Baddeley A. D. Hitch G. J. (1977). Recency re-examined. In Domic S. (Ed.), Attention and performance VI (pp. 647–667). Erlbaum.

[bibr15-17470218241282093] Baddeley A. D. Hitch G. J. (1993). The recency effect: Implicit learning with explicit retrieval? Memory & Cognition, 21, 146–155. 8469122 10.3758/bf03202726

[bibr16-17470218241282093] Baddeley A. D. Hitch G. J. (2019). The phonological loop as a buffer store: An update. Cortex, 112, 91–106. 29941299 10.1016/j.cortex.2018.05.015

[bibr17-17470218241282093] Baddeley A. D. Hitch G. J. Allen R. (2021). A multicomponent model of working memory. In Logie R. H. Camos V. Cowan N. , (Eds.), Working memory. State of science (pp. 1–43). Oxford University Press.

[bibr18-17470218241282093] Baddeley A. D. Lewis V. Vallar G. (1984). Exploring the articulatory loop. Quarterly Journal of Experimental Psychology, 36A, 233–252.

[bibr19-17470218241282093] Baddeley A. D. Thomson N. Buchanan M. (1975). Word length and the structure of short-term memory. Journal of Verbal Learning and Verbal Behavior, 14(6), 575–589.

[bibr20-17470218241282093] Barrouillet P. Gorin S. Camos V. (2021). Simple spans underestimate verbal working memory capacity. Journal of Experimental Psychology: General, 150(4), 633–665. 33017158 10.1037/xge0000957

[bibr21-17470218241282093] Beaman C. P. (2002). Inverting the modality effect in serial recall. The Quarterly Journal of Experimental Psychology Section A, 55, 371–389. 10.1080/02724980143000307 12047050

[bibr22-17470218241282093] Beaman C. P. Jones D. M. (1997). Role of serial order in the irrelevant speech effect: Tests of the changing-state hypothesis. Journal of Experimental Psychology: Learning, Memory, and Cognition, 23(2), 459–471. 10.1037/0278-7393.23.2.459

[bibr23-17470218241282093] Beaman C. P. Jones D. M. (1998). Irrelevant sound disrupts order information in free recall as in serial recall. The Quarterly Journal of Experimental Psychology: Section A, 51, 615–636. 10.1080/713755774 9745380

[bibr24-17470218241282093] Beaman C. P. Morton J. (2000). The separate but related origins of the recency and the modality effect in free recall. Cognition, 77, B59–B65. 10.1016/s0010-0277(00)00107-4 11018512

[bibr25-17470218241282093] Bhatarah P. Ward G. Smith J. Hayes L. (2009). Examining the relationship between free recall and immediate serial recall: Similar patterns of rehearsal and similar effects of word length, presentation rate, and articulatory suppression. Memory & Cognition, 37(5), 689–713. 19487760 10.3758/MC.37.5.689

[bibr26-17470218241282093] Bhatarah P. Ward G. Tan L. (2006). Examining the relationship between free recall and immediate serial recall: The effect of concurrent task performance. Journal of Experimental Psychology. Learning, Memory, and Cognition, 32(2), 215–229. 16569142 10.1037/0278-7393.32.2.215

[bibr27-17470218241282093] Bhatarah P. Ward G. Tan L. (2008). Examining the relationship between free recall and immediate serial recall: The serial nature of recall and the effect of test expectancy. Memory & Cognition, 36, 20–34. 10.3758/MC.36.1.20 18323059

[bibr28-17470218241282093] Brodie D. A. Murdock B. B. (1977). Effect of presentation time on nominal and functional serial-position curves of free recall. Journal of Verbal Learning and Verbal Behavior, 16, 185–200.

[bibr29-17470218241282093] Brown G. D. A. Neath I. Chater N. (2007). A temporal ratio model of memory. Psychological Review, 114(3), 539–576. 17638496 10.1037/0033-295X.114.3.539

[bibr30-17470218241282093] Brown G. D. A. Preece T. Hulme C. (2000). Oscillator-based memory for serial order. Psychological Review, 107, 127–181. 10.1037/0033-295X.107.1.127 10687405

[bibr31-17470218241282093] Burgess N. Hitch G. J. (1992). Toward a network model of the articulatory loop. Journal of Memory and Language, 31, 429–460.

[bibr32-17470218241282093] Burgess N. Hitch G. J. (1999). Memory for serial order: A network model of the phonological loop and its timing. Psychological Review, 106, 551–581.

[bibr33-17470218241282093] Burgess N. Hitch G. J. (2006). A revised model of short-term memory and long-term learning of verbal sequences. Journal of Memory and Language, 55, 627–652. 10.1016/j.jml.2006.08.005

[bibr34-17470218241282093] Campbell R. Dodd B. (1980). Hearing by eye. Quarterly Journal of Experimental Psychology, 32, 85–99. 10.1080/00335558008248235 7367580

[bibr35-17470218241282093] Conrad R. Hull A. J. (1964). Information, acoustic confusion and memory span. British Journal of Psychology, 55(4), 429–432. 14237884 10.1111/j.2044-8295.1964.tb00928.x

[bibr36-17470218241282093] Conway A. Jarrold C. Kane M. Miyake A. Towse J. (Eds.). (2008). Variation in working memory. Oxford University Press.

[bibr37-17470218241282093] Corballis M. C. (1967). Serial order in recognition and recall. Journal of Experimental Psychology, 74(1), 99–105. 6032585 10.1037/h0024500

[bibr38-17470218241282093] Cortis C. Dent K. Kennett S. Ward G. (2015). First things first: Similar list length and output order effects for verbal and nonverbal stimuli. Journal of Experimental Psychology: Learning, Memory, and Cognition, 41(4), 1179–1214. 25528092 10.1037/xlm0000086

[bibr39-17470218241282093] Cowan N. Saults J. S. Elliott E. M. Moreno M. V. (2002). Deconfounding serial recall. Journal of Memory and Language, 46, 153–177.

[bibr40-17470218241282093] Crowder R. G. (1993). Short-term memory: Where do we stand? Memory & Cognition, 21, 142–145. 8469121 10.3758/bf03202725

[bibr41-17470218241282093] Crowder R. G. Morton J. (1969). Precategorical acoustic storage (PAS). Perception & Psychophysics, 5(6), 365–373. 10.3758/BF03210660

[bibr42-17470218241282093] Cyr V. Poirier M. Yearsley J. M. Guitard D. Harrigan I. Saint-Aubin J. (2022). The production effect over the long term: Modeling distinctiveness using serial positions. Journal of Experimental Psychology: Learning, Memory, and Cognition, 48(12), 1797–1820. 34726441 10.1037/xlm0001093

[bibr43-17470218241282093] da Costa Pinto A. Baddeley A. D . (1991). Where did you park your car? Analysis of a naturalistic long-term recency effect. European Journal of Cognitive Psychology, 3, 297–313.

[bibr44-17470218241282093] Dalezman J. J. (1976). Effects of output order on immediate, delayed, and final recall performance. Journal of Experimental Psychology: Human Learning and Memory, 2, 597–608. 10.1037/0278-7393.2.5.597

[bibr45-17470218241282093] Davelaar E. J. Goshen-Gottstein Y. Ashkenazi A. Haarmann H. J. Usher M. (2005). The demise of short-term memory revisited: Empirical and computational investigations of recency effects. Psychological Review, 112(1), 3–42. 15631586 10.1037/0033-295X.112.1.3

[bibr46-17470218241282093] Dougherty M. R. Harbison J. I. Davelaar E. J. (2014). Optional stopping and the termination of memory retrieval. Current Directions in Psychological Science, 23, 332–337. 10.1177/0963721414540170

[bibr47-17470218241282093] Drewnowski A. Murdock B. B. Jr. (1980). The role of auditory features in memory span for words. Journal of Experimental Psychology: Human Learning and Memory, 6, 319–332. 10.1037/0278-7393.6.3.319 7373250

[bibr48-17470218241282093] Ellis N. C. Hennelly R. A. (1980). A bilingual word-length effect: Implications for intelligence testing and the relative ease of mental calculation in Welsh and English. British Journal of Psychology, 71, 43–51.

[bibr49-17470218241282093] Estes W. K. (1955). Statistical theory of spontaneous recovery and regression. Psychological Review, 62(3), 145–154. 14371893 10.1037/h0048509

[bibr50-17470218241282093] Farrell S. A. (2006). Mixed-list phonological similarity effects in delayed serial recall. Journal of Memory and Language, 55, 587–600.

[bibr51-17470218241282093] Farrell S. A. (2012). Temporal clustering and sequencing in short-term memory and episodic memory. Psychological Review, 119(2), 223–271. 22506678 10.1037/a0027371

[bibr52-17470218241282093] Farrell S. A. Lewandowsky S. (2002). An endogenous distributed model of ordering in serial recall. Psychonomic Bulletin & Review, 9, 59–79. 12026954 10.3758/bf03196257

[bibr53-17470218241282093] Farrell S. A. Lewandowsky S. (2008). Empirical and theoretical limits on lag-recency in free recall. Psychonomic Bulletin & Review, 15, 1236–1250. 10.3758/PBR.15.6.1236 19001595

[bibr54-17470218241282093] Gathercole S. E. (Ed.). (1996). Models of short-term memory. Psychology Press.

[bibr55-17470218241282093] Gathercole S. E. (Ed.). (2001). Short-term and working memory (Vol. 9). Psychology Press.

[bibr56-17470218241282093] Gillund G. Shiffrin R. M. (1984). A retrieval model for both recognition and recall. Psychological Review, 91, 1–67. 6571421

[bibr57-17470218241282093] Gionet S. Guitard D. Saint-Aubin J. (2022). The production effect interacts with serial positions. Experimental Psychology, 69(1), 12–22. 35272478 10.1027/1618-3169/a000540 PMC9446468

[bibr58-17470218241282093] Glanzer M. (1972). Storage mechanisms in recall. In Bower G. H. (Ed.), The psychology of learning and motivation: Advances in research and theory (Vol. V, pp. 129–193). Academic Press.

[bibr59-17470218241282093] Glanzer M. Cunitz A. R. (1966). Two storage mechanisms in free recall. Journal of Verbal Learning and Verbal Behavior, 5, 351–360.

[bibr60-17470218241282093] Glenberg A. M. (1984). A retrieval account of the long-term modality. Cognition, 10, 16–31. 10.1037//0278-7393.10.1.16 6242733

[bibr61-17470218241282093] Glenberg A. M. (1987). Temporal context and recency. In Gorfein D. S. Hoffman R. R. (Eds.), Memory and learning: The Ebbinghaus centennial conference (pp. 173–190). Lawrence Erlbaum.

[bibr62-17470218241282093] Glenberg A. M. Swanson N. G. (1986). A temporal distinctiveness theory of recency and modality effects. Journal of Experimental Psychology: Learning, Memory and Cognition, 12, 3–15. 2949048 10.1037//0278-7393.12.1.3

[bibr63-17470218241282093] Golomb J. D. Peelle J. E. Addis K. M. Kahana M. J. Wingfield A. (2008). Effects of adult aging on utilization of temporal and semantic associations during free and serial recall. Memory & Cognition, 36, 947–956. 10.3758/MC.36.5.947 18630201 PMC2839458

[bibr64-17470218241282093] Greene R. L. (1992). Human memory: Paradigms and paradoxes. Psychology Press.

[bibr65-17470218241282093] Greene R. L. Samuel A. G. (1986). Recency and suffix effects in serial recall of musical stimuli. Journal of Experimental Psychology: Learning, Memory, and Cognition, 12, 517–524. 10.1037/0278-7393.12.4.517 2945898

[bibr66-17470218241282093] Grenfell-Essam R. Ward G. (2012). Examining the relationship between free recall and immediate serial recall: The role of list length, strategy use, and test expectancy. Journal of Memory and Language, 67(1), 106–148.

[bibr67-17470218241282093] Grenfell-Essam R. Ward G. Cortis Mack C. (2019). Temporal isolation effects in immediate recall. Journal of Memory and Language, 109, 104049.

[bibr68-17470218241282093] Grenfell-Essam R. Ward G. Tan L. (2013). The role of rehearsal on the output order of immediate free recall of short and long lists. Journal of Experimental Psychology: Learning, Memory, and Cognition, 39(2), 317–347. 22774850 10.1037/a0028974

[bibr69-17470218241282093] Grenfell-Essam R. Ward G. Tan L. (2017). Common modality effects in immediate free recall and immediate serial recall. Journal of Experimental Psychology. Learning, Memory, and Cognition, 43, 1909–1933. 28557502 10.1037/xlm0000430 PMC5729966

[bibr70-17470218241282093] Grossberg S. Pearson L. R. (2008). Laminar cortical dynamics of cognitive and motor working memory, sequence learning and performance: Toward a unified theory of how the cerebral cortex works. Psychological Review, 115, 677–732. 18729596 10.1037/a0012618

[bibr71-17470218241282093] Hartley T. Hurlstone M. J. Hitch G. J. (2016). Effects of rhythm on memory for spoken sequences: A model and tests of its stimulus-driven mechanism. Cognitive Psychology, 87, 135–178. 27261540 10.1016/j.cogpsych.2016.05.001

[bibr72-17470218241282093] Healey M. K. Kahana M. J. (2016). A Four-component model of age-related memory change. Psychological Review, 123(1), 23–69. 26501233 10.1037/rev0000015 PMC5067022

[bibr73-17470218241282093] Healey M. K. Long N. M. Kahana M. J. (2019). Contiguity in episodic memory. Psychonomic Bulletin & Review, 26(3), 699–720. 30465268 10.3758/s13423-018-1537-3 PMC6529295

[bibr74-17470218241282093] Healey M. K. Wahlheim C. N. (2024). PEPPR: A post-encoding pre-production reinstatement model of dual-list free recall. Memory & Cognition, 52(1), 163–181. 37782445 10.3758/s13421-023-01453-z

[bibr75-17470218241282093] Hebb D. O. (1961). Distinctive features of learning in the higher animal. In Delafresnaye J. F. (Ed.), Brain mechanisms and learning (pp. 37–46). Oxford: Blackwell.

[bibr76-17470218241282093] Henson R. N. A. (1998). Short-term memory for serial order: The start-end model of serial recall. Cognitive Psychology, 36, 73–137. 9721198 10.1006/cogp.1998.0685

[bibr77-17470218241282093] Henson R. N. A. (1999). Positional information in short-term memory: Relative or absolute? Memory & Cognition, 27, 915–927. 10540820 10.3758/bf03198544

[bibr78-17470218241282093] Henson R. N. A. (2001). Serial order in short-term memory. The Psychologist, 14(2), 70–73.

[bibr79-17470218241282093] Henson R. N. A. Norris D. G. Page M. P. A. Baddeley A. D. (1996). Unchained memory: Error patterns rule out chaining models of immediate serial recall. The Quarterly Journal of Experimental Psychology A: Human Experimental Psychology, 49A(1), 80–115. 10.1080/027249896392810

[bibr80-17470218241282093] Hitch G. J. Ferguson J. (1991). Prospective memory for future intentions: Some comparisons with memory for past events. European Journal of Cognitive Psychology, 3(3), 285–295.

[bibr81-17470218241282093] Howard M. W. Kahana M. J. (1999). Contextual variability and serial position effects in free recall. Journal of Experimental Psychology. Learning, Memory, and Cognition, 25(4), 923–941. 10439501 10.1037//0278-7393.25.4.923

[bibr82-17470218241282093] Howard M. W. Kahana M. J. (2002). A distributed representation of temporal context. Journal of Mathematical Psychology, 46(3), 269–299.

[bibr83-17470218241282093] Hughes R. W. (2024). The phonological store of working memory: A critique and an alternative, perceptual-motor, approach to verbal short-term memory. Quarterly Journal of Experimental Psychology, 0(0). 10.1177/17470218241257885 PMC11783984 38785305

[bibr84-17470218241282093] Hulme C. Maughan S. Brown G. D. (1991). Memory for familiar and unfamiliar words: Evidence for a long-term memory contribution to short-term memory span. Journal of Memory and Language, 30(6), 685–701.

[bibr85-17470218241282093] Hulme C. Roodenrys S. Schweickert R. Brown G. D. Martin S. Stuart G. (1997). Word-frequency effects on short-term memory tasks: Evidence for a redintegration process in immediate serial recall. Journal of Experimental Psychology: Learning, Memory, and Cognition, 23(5), 1217–1232. 9293631 10.1037//0278-7393.23.5.1217

[bibr86-17470218241282093] Hulme C. Thomson N. Muir C. Lawrence A. (1984). Speech rate and the development of short-term memory span. Journal of Experimental Child Psychology, 38(2), 241–253. 10.1006/jecp.1993.1043 8301247

[bibr87-17470218241282093] Hurlstone M. J. (2024). Serial recall. In Kahana M. Wagner A. (Eds.), The Oxford handbook on human memory. (pp. 799–830) Oxford University Press.

[bibr88-17470218241282093] Hurlstone M. J. Hitch G. J. Baddeley A. D. (2014). Memory for serial order across domains: An overview of the literature and directions for future research. Psychological Bulletin, 140, 339–373. 24079725 10.1037/a0034221

[bibr89-17470218241282093] Jahnke J. C. (1963). Serial position effects in immediate serial recall. Journal of Verbal Learning and Verbal Behavior, 2(3), 284–287.

[bibr90-17470218241282093] Jarrold C. (2017). The Mid-Career Award: Working out how working memory works: Evidence from typical and atypical development. Quarterly Journal of Experimental Psychology, 70(9), 1747–1767. 10.1080/17470218.2016.1213869 27434538

[bibr91-17470218241282093] Jones D. M. Farrand P. Stuart G. Morris N. (1995). Functional equivalence of verbal and spatial information in serial short-term memory. Journal of Experimental Psychology: Learning, Memory, and Cognition, 21(4), 1008–1018. 7673864 10.1037//0278-7393.21.4.1008

[bibr92-17470218241282093] Jones D. M. Hughes R. W. Macken W. J. (2006). Perceptual organization masquerading as phonological storage: Further support for a perceptual-gestural view of short-term memory. Journal of Memory and Language, 54(2), 265–281.

[bibr93-17470218241282093] Jones D. M. Macken B. (2018). In the beginning was the deed: Verbal short-term memory as object-oriented action. Current Directions in Psychological Science, 27(5), 351–356.

[bibr94-17470218241282093] Jones D. M. Macken W. J. Nicholls A. P. (2004). The phonological store of working memory: Is it phonological and is it a store? Journal of Experimental Psychology: Learning, Memory, and Cognition, 30(3), 656–674. 10.1037/0278-7393.30.3.656 15099134

[bibr95-17470218241282093] Jones G. Macken B. (2018). Long-term associative learning predicts verbal short-term memory performance. Memory & Cognition, 46, 216–229. 28971367 10.3758/s13421-017-0759-3 PMC5809536

[bibr96-17470218241282093] Kahana M. J. (1996). Associative retrieval processes in free recall. Memory & Cognition, 24, 103–109. 8822162 10.3758/bf03197276

[bibr97-17470218241282093] Kahana M. J. (2020). Computational models of memory search. Annual Review of Psychology, 71, 107–138. 10.1146/annurev-psych-010418-103358 PMC8389167 31567043

[bibr98-17470218241282093] Kahana M. J. Diamond N. B. Aka A. (2024). Laws of human memory. In Kahana M. J. Wagner A. D. (Eds.), Oxford handbook of human memory (pp. 29–63). Oxford University Press.

[bibr99-17470218241282093] Kahana M. J. Mollison M. V. Addis K. M. (2010). Positional cues in serial learning: The spin list technique. Memory & Cognition, 38(1), 92–101. 19966242 10.3758/MC.38.1.92 PMC2839411

[bibr100-17470218241282093] Klein K. A. Addis K. M. Kahana M. J. (2005). A comparative analysis of serial and free recall. Memory & Cognition, 33, 833–839. 16383171 10.3758/bf03193078

[bibr101-17470218241282093] Kragel J. E. Morton N. W. Polyn S. M. (2015). Neural activity in the medial temporal lobe reveals the fidelity of mental time travel. Journal of Neuroscience, 35(7), 2914–2926. 25698731 10.1523/JNEUROSCI.3378-14.2015 PMC6605592

[bibr102-17470218241282093] Laming D. (1999). Testing the idea of distinct storage mechanisms in memory. International Journal of Psychology, 34, 419–426.

[bibr103-17470218241282093] Laming D. (2008). An improved algorithm for predicting free recalls. Cognitive Psychology, 57(3), 179–219. 18329010 10.1016/j.cogpsych.2008.01.001

[bibr104-17470218241282093] Laming D. (2009). Failure to recall. Psychological Review, 116(1), 157–186. 19159152 10.1037/a0014150

[bibr105-17470218241282093] Laming D. (2010). Serial position curves in free recall. Psychological Review, 117(1), 93–133. 20063965 10.1037/a0017839

[bibr106-17470218241282093] Lehman M. Malmberg K. J. (2013). A buffer model of memory encoding and temporal correlations in retrieval. Psychological Review, 120(1), 155–189. 23230891 10.1037/a0030851

[bibr107-17470218241282093] Lewandowsky S. Brown G. D. A. Thomas J. L. (2009). Traveling economically through memory space: Characterizing output order in memory for serial order. Memory & Cognition, 37, 181–193. 19223568 10.3758/MC.37.2.181

[bibr108-17470218241282093] Lewandowsky S. Farrell S. (2008). Short-term memory: New data and a model. The Psychology of Learning and Motivation, 49, 1–48. 10.1016/S0079-7421(08)00001-7

[bibr109-17470218241282093] Lewandowsky S. Murdock B. B. Jr. (1989). Memory for serial order. Psychological Review, 96(1), 25–57.

[bibr110-17470218241282093] Lewandowsky S. Oberauer K. (2015). Rehearsal in serial recall: An unworkable solution to the nonexistent problem of decay. Psychological Review, 122(4), 674–699. 26437148 10.1037/a0039684

[bibr111-17470218241282093] Li S. C. Lewandowsky S. (1995). Forward and backward recall: Different retrieval processes. Journal of Experimental Psychology: Learning, Memory, and Cognition, 21(4), 837–847.

[bibr112-17470218241282093] Loaiza V. M. McCabe D. P. (2012). Temporal–contextual processing in working memory: Evidence from delayed cued recall and delayed free recall tests. Memory & Cognition, 40(2), 191–203. 10.3758/s13421-011-0148-2 21948350

[bibr113-17470218241282093] Logan G. D. (2021). Serial order in perception, memory, and action. Psychological Review, 128(1), 1–44. 32804525 10.1037/rev0000253

[bibr114-17470218241282093] Logan G. D. Cox G. E. (2021). Serial memory: Putting chains and position codes in context. Psychological Review, 128(6), 1197–1205. 10.1037/rev0000327 34570522

[bibr115-17470218241282093] Logan G. D. Cox G. E. (2023). Serial order depends on item-dependent and item-independent contexts. Psychological Review, 130(6), 1672–1687. 36892899 10.1037/rev0000422

[bibr116-17470218241282093] Lohnas L. J. (2023, December 31). A retrieved context model of serial recall and free recall. 10.31234/osf.io/9t4y6 PMC13298665 42459226

[bibr117-17470218241282093] Lohnas L. J. Polyn S. M. Kahana M. J. (2015). Expanding the scope of memory search: Modeling intralist and interlist effects in free recall. Psychological Review, 122(2), 337–363. 25844876 10.1037/a0039036

[bibr118-17470218241282093] Macken B. Taylor J. C. Kozlov M. D. Hughes R. W. Jones D. M. (2016). Memory as embodiment: The case of modality and serial short-term memory. Cognition, 155, 113–124. 27376662 10.1016/j.cognition.2016.06.013

[bibr119-17470218241282093] McCabe D. P. (2008). The role of covert retrieval in working memory span tasks: Evidence from delayed recall tests. Journal of Memory and Language, 58(2), 480–494. 19633737 10.1016/j.jml.2007.04.004 PMC2715014

[bibr120-17470218241282093] Mensink G.-J. M. Raaijmakers J. G. (1988). A model for interference and forgetting. Psychological Review, 95(4), 434–455.

[bibr121-17470218241282093] Mensink G.-J. M. Raaijmakers J. G. (1989). A model for contextual fluctuation. Journal of Mathematical Psychology, 33(2), 172–186.

[bibr122-17470218241282093] Metcalfe J. Murdock B. B. (1981). An encoding and retrieval model of single-trial free recall. Journal of Verbal Learning and Verbal Behavior, 20(2), 161–189.

[bibr123-17470218241282093] Miyake A. Shah P. (Eds.). (1999). Models of working memory. Cambridge University Press.

[bibr124-17470218241282093] Modigliani V. Hedges D. G. (1987). Distributed rehearsals and the primacy effect in single-trial free recall. Journal of Experimental Psychology: Learning, Memory, and Cognition, 13, 426–436.

[bibr125-17470218241282093] Morton N. W. Polyn S. M. (2016). A predictive framework for evaluating models of semantic organization in free recall. Journal of Memory and Language, 86, 119–140. 28331243 10.1016/j.jml.2015.10.002 PMC5358688

[bibr126-17470218241282093] Murdock B. B. Jr. (1962). The serial position effect of free recall. Journal of Experimental Psychology, 64(5), 482–488.

[bibr127-17470218241282093] Murdock B. B. Jr. (1993). TODAM2: A model for the storage and retrieval of item, associative, and serial-order information. Psychological Review, 100(2), 183–203. 10.1037/0033-295X.100.2.183 8483981

[bibr128-17470218241282093] Murdock B. B. Jr. (1995). Developing TODAM: Three models for serial-order information. Memory & Cognition, 23(5), 631–645. 7476248 10.3758/bf03197264

[bibr129-17470218241282093] Murdock B. B. Jr. Walker K. D. (1969). Modality effects in free recall. Journal of Verbal Learning and Verbal Behavior, 8(5), 665–676.

[bibr130-17470218241282093] Murray A. Jones D. M. (2002). Articulatory complexity at item boundaries in serial recall: The case of Welsh and English digit span. Journal of Experimental Psychology: Learning, Memory, and Cognition, 28(3), 594–598. 10.1037/0278-7393.28.3.594 12018511

[bibr131-17470218241282093] Nairne J. S. (1988). A framework for interpreting recency effects in immediate serial recall. Memory & Cognition, 16, 343–352. 3062314 10.3758/bf03197045

[bibr132-17470218241282093] Nairne J. S. (1990). A feature model of immediate memory. Memory & Cognition, 18, 251–269. 2192233 10.3758/bf03213879

[bibr133-17470218241282093] Nairne J. S. (2002). Remembering over the short-term: The case against the standard model. Annual Review of Psychology, 53(1), 53–81. 10.1146/annurev.psych.53.100901.135131 11752479

[bibr134-17470218241282093] Naveh-Benjamin M. Ayres T. J. (1986). Digit span, reading rate, and linguistic relativity. Quarterly Journal of Experimental Psychology, 38(A), 739–751. 10.1080/14640748608401623 3809578

[bibr135-17470218241282093] Neath I. (1999). Modelling the disruptive effects of irrelevant speech on order information. International Journal of Psychology, 34(5–6), 410–418.

[bibr136-17470218241282093] Neath I. (2000). Modeling the effects of irrelevant speech on memory. Psychonomic Bulletin & Review, 7, 403–423. 11082850 10.3758/bf03214356

[bibr137-17470218241282093] Neath I. Crowder R. G. (1996). Distinctiveness and very short-term serial position effects. Memory, 4, 225–242. 10.1080/096582196388933 8735609

[bibr138-17470218241282093] Neath I. Nairne J. S. (1995). Word-length effects in immediate memory: Overwriting trace decay theory. Psychonomic Bulletin & Review, 2(4), 429–441. 24203783 10.3758/BF03210981

[bibr139-17470218241282093] Oberauer K. (2003). Understanding serial position curves in short-term recognition and recall. Journal of Memory and Language, 49(4), 469–483.

[bibr140-17470218241282093] Oberauer K. (2019). Is rehearsal an effective maintenance strategy for working memory? Trends in Cognitive Sciences, 23(9), 798–809. 10.1016/j.tics.2019.06.002 31301953

[bibr141-17470218241282093] Oberauer K. (2022). When does working memory get better with longer time? Journal of Experimental Psychology. Learning, Memory, and Cognition, 48(12), 1754–1774. 36326652 10.1037/xlm0001199

[bibr142-17470218241282093] Oberauer K. Lewandowsky S. (2008). Forgetting in immediate serial recall: Decay, temporal distinctiveness, or interference? Psychological Review, 115, 544–576. 10.1037/0033-295X.115.3.544 18729591

[bibr143-17470218241282093] Osth A. F. Dennis S. (2015). The fill-in effect in serial recall can be obscured by omission errors. Journal of Experimental Psychology. Learning, Memory, and Cognition, 41(5), 1447–1455. 25893843 10.1037/xlm0000113

[bibr144-17470218241282093] Osth A. F. Farrell S. (2019). Using response time distributions and race models to characterize primacy and recency effects in free recall initiation. Psychological Review, 126(4), 578–609. 30998031 10.1037/rev0000149

[bibr145-17470218241282093] Osth A. F. Hurlstone M. J. (2023). Do item-dependent context representations underlie serial order in cognition? Commentary on Logan (2021). Psychological Review, 130, 513–545. 35099212 10.1037/rev0000352

[bibr146-17470218241282093] Osth A. F. Reed A. Farrell S. (2021). How do recall requirements affect decision-making in free recall initiation? A linear ballistic accumulator approach. Memory & Cognition, 49, 968–983. 33528805 10.3758/s13421-020-01117-2 PMC7852469

[bibr147-17470218241282093] Page M. P. A. Norris D. (1998). The primacy model: A new model of immediate serial recall. Psychological Review, 105, 761–781. 9830378 10.1037/0033-295x.105.4.761-781

[bibr148-17470218241282093] Pazdera J. K. Kahana M. J. (2023). Modality effects in free recall: A retrieved-context account. Journal of Experimental Psychology. Learning, Memory, and Cognition, 49(6), 866–888. 35787136 10.1037/xlm0001140

[bibr149-17470218241282093] Poirier M. Saint-Aubin J. (1995). Memory for related and unrelated words: Further evidence on the influence of semantic factors in immediate serial recall. The Quarterly Journal of Experimental Psychology A: Human Experimental Psychology, 48A(2), 384–404. 10.1080/14640749508401396 7610273

[bibr150-17470218241282093] Polyn S. M. Norman K. A. Kahana M. J. (2009). A context maintenance and retrieval model of organizational processes in free recall. Psychological Review, 116(1), 129–156. 19159151 10.1037/a0014420 PMC2630591

[bibr151-17470218241282093] Postman L. Phillips L. W. (1965). Short-term temporal changes in free recall. Quarterly Journal of Experimental Psychology, 17, 132–138.

[bibr152-17470218241282093] Raaijmakers J. G. W. Shiffrin R. M. (1981). Search of associative memory. Psychological Review, 88, 93–134.

[bibr153-17470218241282093] Robbins T. W. Anderson E. J. Barker D. R. Bradley A. C. Fearnyhough C. Henson R. Hudson S. R. Baddeley A. D. (1996). Working memory in chess. Memory & Cognition, 24, 83–93. 8822160 10.3758/bf03197274

[bibr154-17470218241282093] Roberts W. A. (1972). Free recall of word lists varying in length and rate of presentation: A test of total-time hypotheses. Journal of Experimental Psychology, 92(3), 365–372.

[bibr155-17470218241282093] Roodenrys S. Hulme C. Lethbridge A. Hinton M. Nimmo L. M. (2002). Word-frequency and phonological-neighborhood effects on verbal short-term memory. Journal of Experimental Psychology: Learning, Memory, and Cognition, 28(6), 1019–1034. 12450329 10.1037//0278-7393.28.6.1019

[bibr156-17470218241282093] Rundus D. (1971). Analysis of rehearsal processes in free recall. Journal of Experimental Psychology, 89, 63–77.

[bibr157-17470218241282093] Saint-Aubin J. Poirier M. (1999). Semantic similarity and immediate serial recall: Is there a detrimental effect on order information? The Quarterly Journal of Experimental Psychology A: Human Experimental Psychology, 52A(2), 367–394. 10.1080/713755814 10428684

[bibr158-17470218241282093] Saint-Aubin J. Poirier M. Yearsley J. Robichaud J. M. Guitard D. (2023). Modeling verbal short-term memory: A walk around the neighborhood. Journal of Experimental Psychology: Learning, Memory, and Cognition, 49(2), 198–215. 36996187 10.1037/xlm0001226

[bibr159-17470218241282093] Saint-Aubin J. Yearsley J. M. Poirier M. Cyr V. Guitard D. (2021). A model of the production effect over the short-term: The cost of relative distinctiveness. Journal of Memory and Language, 118, 104219.

[bibr160-17470218241282093] Schweickert R. Boruff B. (1986). Short-term memory capacity: Magic number or magic spell? Journal of Experimental Psychology: Learning, Memory, and Cognition, 12(3), 419–425. 2942626 10.1037//0278-7393.12.3.419

[bibr161-17470218241282093] Sederberg P. B. Howard M. W. Kahana M. J. (2008). A context-based theory of recency and contiguity in free recall. Psychological Review, 115, 893–912. 10.1037/a0013396 18954208 PMC2585999

[bibr162-17470218241282093] Solway A. Murdock B. B. Kahana M. J. (2012). Positional and temporal clustering in serial order memory. Memory & Cognition, 40, 177–190. 22057363 10.3758/s13421-011-0142-8 PMC3282556

[bibr163-17470218241282093] Souza A. S. Oberauer K. (2018). Does articulatory rehearsal help immediate serial recall? Cognitive Psychology, 107, 1–21. 30292953 10.1016/j.cogpsych.2018.09.002

[bibr164-17470218241282093] Souza A. S. Oberauer K. (2020). No evidence that articulatory rehearsal improves complex span performance. Journal of Cognition, 3(1), 11–11. 32435749 10.5334/joc.103 PMC7227395

[bibr165-17470218241282093] Spurgeon J. Ward G. Matthews W. J. (2014). Examining the relationship between immediate serial recall and immediate free recall: Common effects of phonological loop variables but only limited evidence for the phonological loop. Journal of Experimental Psychology: Learning, Memory, & Cognition, 40, 1110–1141. 10.1037/a0035784 24564540

[bibr166-17470218241282093] Spurgeon J. Ward G. Matthews W. J. Farrell S. (2015). Can the effects of temporal grouping explain the similarities and differences between free recall and serial recall? Memory & Cognition, 43, 469–488. 25331276 10.3758/s13421-014-0471-5

[bibr167-17470218241282093] Standing L. Bond B. Smith P. Isely C. (1980). Is the immediate memory span determined by subvocalisation rate? British Journal of Psychology, 71, 525–539.

[bibr168-17470218241282093] Tan L. Ward G. (2000). A recency-based account of the primacy effect in free recall. Journal of Experimental Psychology: Learning, Memory, & Cognition, 26, 1589–1625. 11185785 10.1037//0278-7393.26.6.1589

[bibr169-17470218241282093] Tan L. Ward G. (2007). Output order in immediate serial recall. Memory & Cognition, 35, 1093–1106. 17910192 10.3758/bf03193481

[bibr170-17470218241282093] Tan L. Ward G. (2008). Rehearsal in immediate serial recall. Psychonomic Bulletin & Review, 15, 535–542. http://dx.doi.org/10.3758/PBR.15.3.535 18567251 10.3758/pbr.15.3.535

[bibr171-17470218241282093] Tulving E. (1968). Theoretical issues in free recall. In Dixon T. R. Horton D. L. (Eds.), Verbal behavior and general behavior theory (pp. 2–36). Prentice-Hall.

[bibr172-17470218241282093] Unsworth N. Engle R. W. (2007). The nature of individual differences in working memory capacity: Active maintenance in primary memory and controlled search from secondary memory. Psychological Review, 114, 104–132. 10.1037/0033-295X.114.1.104 17227183

[bibr173-17470218241282093] Walker I. Hulme C. (1999). Concrete words are easier to recall than abstract words: Evidence for a semantic contribution to short-term serial recall. Journal of Experimental Psychology: Learning, Memory, and Cognition, 25(5), 1256–1271.

[bibr174-17470218241282093] Ward G. (2001). A critique of the working memory model. In Andrade J. (Ed.), Working memory in perspective (pp. 219–239). Psychology Press.

[bibr175-17470218241282093] Ward G. (2024). Rehearsal processes. In Kahana M. J. Wagner A. D. (Eds.), Oxford handbook of human memory (pp. 614–649). Oxford University Press.

[bibr176-17470218241282093] Ward G. Avons S. E. Melling L. (2005). Serial position curves in short-term memory: Functional equivalence across modalities. Memory, 13(3–4), 308–317. 15948615 10.1080/09658210344000279

[bibr177-17470218241282093] Ward G. Tan L. (2019). Control processes in short-term storage: Retrieval strategies in immediate recall depend upon the number of words to be recalled. Memory & Cognition, 47, 658–682. 10.3758/s13421-018-0891-8 30617748

[bibr178-17470218241282093] Ward G. Tan L. (2023). The role of rehearsal and reminding in the recall of categorized word lists. Cognitive Psychology, 143, 101563. 37141672 10.1016/j.cogpsych.2023.101563

[bibr179-17470218241282093] Ward G. Tan L. Grenfell-Essam R. (2010). Examining the relationship between free recall and immediate serial recall: The effects of list length and output order. Journal of Experimental Psychology: Learning, Memory, and Cognition, 36(5), 1207–1241. 20804293 10.1037/a0020122

[bibr180-17470218241282093] Waugh N. C. (1961). Free versus serial recall. Journal of Experimental Psychology, 62, 496–502. 14005352 10.1037/h0043891

