# Supplementary material for: The Working Memory Model and the relationship between immediate serial recall and immediate free recall
Source: Q J Exp Psychol (Hove). 2024 Nov 1;78(2):310–36. doi: 10.1177/17470218241282093 (PMC11783987; doi:10.1177/17470218241282093)
Supplement: sj-docx-1-qjp-10.1177_17470218241282093 – Supplemental material for The Working Memory Model and the relationship between immediate serial recall and immediate free recall [file sj-docx-1-qjp-10.1177_17470218241282093.docx]

Supplementary Material for:

**The Working Memory Model and the relationship between immediate serial recall and immediate free recall**

Geoff Ward^1^ and C. Philip Beaman^2^

^1^Department of Psychology, University of Essex

^2^School of Psychology and Clinical Language Sciences, University of Reading

**Author note**

We have no known conflicts of interest to disclose. The novel data analyses are from published data – the raw data can be found here:

Ward and Grenfell-Essam (2012, JML): http://dx.doi.org/10.1016/j.jml.2012.04.004

Osth and Dennis (2015, JEPLM&C): <https://osf.io/8zycm/>

Correspondence concerning this article should be sent to:

Geoff Ward
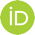
 [https://orcid.org/0000-0002-7309-1404](https://linkprotect.cudasvc.com/url?a=https%3a%2f%2forcid.org%2f0000-0002-7309-1404%3flang%3den&c=E,1,Q9MM9LSqVeFnmq6n90bTLVRy8NLFT5oQfzECLivElSXzyvKHv5xwb_j6Ft-6jwViT2qZhs3Dinvp7nE703ewiEzDff8ZDO8onUw-2N_0AfyOGb-c&typo=1) Department of Psychology, University of Essex, Wivenhoe Park, Colchester, CO4 3SQ, UK.

Email: [gdward@essex.ac.uk](mailto:gdward@essex.ac.uk) or

Philip Beaman, School of Psychology & Clinical Language Sciences, Harry Pitt Building, University of Reading, Earley Gate, Reading RG6 6ES, UK.

Email: [c.p.beaman@reading.ac.uk](mailto:c.p.beaman@reading.ac.uk)

Supplementary Material A1. Mean length of start-sequence for each task, condition, and list length. A start-sequence refers to any forward-ordered run of recalls, beginning with the first presented item.

|  |  |  | List Length | | | | | | | | | | |  |  |
| --- | --- | --- | --- | --- | --- | --- | --- | --- | --- | --- | --- | --- | --- | --- | --- |
| Sequence | Task |  | 1 | 2 | 3 | 4 | 5 | 6 | 7 | 8 | 10 | 12 | 15 | *Mean* | Data Source |
| Start-seq. | ISR | Pre-cued |  | 1.994 |  | 3.106 | 1.944 | 1.256 | 0.819 | 0.788 |  | 0.419 | 0.225 | *1.319* | G-E&W(2012) |
|  |  | Post-cued |  | 1.994 |  | 2.819 | 1.881 | 1.206 | 0.775 | 0.656 |  | 0.313 | 0.325 | *1.246* | G-E&W(2012) |
|  |  | Visual silent | 0.990 | 2.000 | 2.833 | 3.177 | 3.052 | 2.469 | 2.104 | 1.667 | 1.573 | 1.385 | 1.083 | *2.030* | SW&M(2014) |
|  |  | Visual AS | 1.021 | 1.896 | 1.906 | 1.708 | 1.188 | 1.229 | 1.042 | 0.708 | 0.740 | 0.792 | 0.615 | *1.168* | SW&M(2014) |
|  |  | Short words |  |  |  |  |  | 2.650 |  |  |  |  |  | *2.650* | BWS&H(2009) |
|  |  | Medium words |  |  |  |  |  | 1.883 |  |  |  |  |  | *1.883* | BWS&H(2009) |
|  |  | Long words |  |  |  |  |  | 0.675 |  |  |  |  |  | *0.683* | BWS&H(2009) |
|  |  |  |  |  |  |  |  |  |  |  |  |  |  |  |  |
|  | IFR | Pre-cued |  | 1.975 |  | 2.850 | 1.906 | 1.119 | 0.706 | 0.563 |  | 0.363 | 0.344 | *1.228* | G-E&W(2012) |
|  |  | Post-cued |  | 1.956 |  | 2.838 | 1.850 | 1.156 | 0.756 | 0.588 |  | 0.338 | 0.331 | *1.227* | G-E&W(2012) |
|  |  | Visual Silent | 1.000 | 1.927 | 2.625 | 2.708 | 2.333 | 1.583 | 1.229 | 0.656 | 0.625 | 0.604 | 0.708 | *1.455* | SW&M(2014) |
|  |  | Visual AS | 1.000 | 1.656 | 1.563 | 1.073 | 0.792 | 0.427 | 0.281 | 0.240 | 0.240 | 0.198 | 0.104 | *0.688* | SW&M(2014) |
|  |  |  |  |  |  |  |  |  |  |  |  |  |  |  |  |
|  |  | Short words |  |  |  |  |  | 1.675 |  |  |  |  |  | *1.675* | BWS&H(2009) |
|  |  | Medium words |  |  |  |  |  | 1.117 |  |  |  |  |  | *1.117* | BWS&H(2009) |
|  |  | Long words |  |  |  |  |  | 0.325 |  |  |  |  |  | *0.325* | BWS&H(2009) |
|  |  |  |  |  |  |  |  |  |  |  |  |  |  |  |  |

Note: G-E&W(2012) refers to Grenfell-Essam and Ward (2012, E3) and the manipulation of when the task is known prior or after encoding. SW&M(2014) refers to Spurgeon, Ward and Matthews (2014, E1), and the manipulation is articulatory suppression (AS). BWS&H(2009) refers to Bhatarah, Ward, Smith and Hayes (2009, E4) and the manipulation of word length. IFR refers to immediate free recall and ISR refers to Immediate Serial Recall.

Supplementary Material A2. Mean length of end-sequence for each task, condition, and list length. An end-sequence refers to any forward-ordered run of recalls, terminating with the last presented item.

|  |  |  | List Length | | | | | | | | | | | |  |  | |
| --- | --- | --- | --- | --- | --- | --- | --- | --- | --- | --- | --- | --- | --- | --- | --- | --- | --- |
| Sequence | Task |  | 1 | 2 | 3 | 4 | 5 | 6 | 7 | 8 | 10 | 12 | 15 |  | *Mean* Data Source | |  |
| End -seq. | ISR | Pre-cued |  | 0.000 |  | 0.375 | 0.894 | 1.169 | 1.219 | 1.281 |  | 1.613 | 1.550 |  | *1.013* G-E&W(2012) | |  |
|  |  | Post-cued |  | 0.000 |  | 0.400 | 1.000 | 1.269 | 1.294 | 1.375 |  | 1.538 | 1.538 |  | *1.052* G-E&W(2012) | |  |
|  |  | Visual silent | 0.000 | 0.000 | 0.042 | 0.250 | 0.406 | 0.531 | 0.615 | 0.510 | 0.833 | 0.813 | 0.750 |  | *0.432* SW&M(2014) | |  |
|  |  | Visual AS | 0.000 | 0.042 | 0.385 | 0.490 | 0.594 | 0.688 | 0.646 | 0.625 | 0.750 | 0.604 | 0.667 |  | *0.499* SW&M(2014) | |  |
|  |  | Short words |  |  |  |  |  | 0.792 |  |  |  |  |  |  | *0.792* BWS&H(2009) | |  |
|  |  | Medium words |  |  |  |  |  | 0.817 |  |  |  |  |  |  | *0.817* BWS&H(2009) | |  |
|  |  | Long words |  |  |  |  |  | 0.658 |  |  |  |  |  |  | *0.658* BWS&H(2009) | |  |
|  |  |  |  |  |  |  |  |  |  |  |  |  |  |  |  | |  |
|  | IFR | Pre-cued |  | 0.006 |  | 0.506 | 1.038 | 1.188 | 1.556 | 1.456 |  | 1.500 | 1.431 |  | *1.085*  G-E&W(2012) | |  |
|  |  | Post-cued |  | 0.038 |  | 0.438 | 1.113 | 1.225 | 1.263 | 1.325 |  | 1.394 | 1.363 |  | *1.020* G-E&W(2012) | |  |
|  |  | Visual Silent | 0.000 | 0.031 | 0.156 | 0.365 | 0.688 | 0.740 | 0.677 | 0.948 | 0.854 | 0.885 | 0.813 |  | *0.560* SW&M(2014) | |  |
|  |  | Visual AS | 0.000 | 0.156 | 0.521 | 0.750 | 0.792 | 0.990 | 0.896 | 0.875 | 0.792 | 0.750 | 0.750 |  | *0.661* SW&M(2014) | |  |
|  |  | Short words |  |  |  |  |  | 1.217 |  |  |  |  |  |  | *1.217* BWS&H(2009) | |  |
|  |  | Medium words |  |  |  |  |  | 1.208 |  |  |  |  |  |  | *1.208* BWS&H(2009) | |  |
|  |  | Long words |  |  |  |  |  | 1.108 |  |  |  |  |  |  | *1.108* BWS&H(2009) | |  |
|  |  |  |  |  |  |  |  |  |  |  |  |  |  |  |  |  |  |
|  |  |  |  |  |  |  |  |  |  |  |  |  |  |  |  |  |  |

Note: G-E&W(2012) refers to Grenfell-Essam and Ward (2012, E3) and the manipulation of when the task is known prior or after encoding. SW&M(2014) refers to Spurgeon, Ward and Matthews (2014, E1), and the manipulation is articulatory suppression (AS). BWS&H(2009) refers to Bhatarah, Ward, Smith and Hayes (2009, E4) and the manipulation of word length. IFR refers to immediate free recall and ISR refers to Immediate Serial Recall.
